# Neuron-intrinsic and glial pathways regulate sensory cilia regeneration in adult *C. elegans*

**DOI:** 10.64898/2026.05.15.725580

**Published:** 2026-05-17

**Authors:** Kirsten Judge, Alison Philbrook, Stephen Nurrish, Samantha Leslie, Laura Grunenkovaite, Yu-Ming Lu, Piali Sengupta

**Affiliations:** 1 Department of Biology, Brandeis University, Waltham, MA 02454, USA; 2 Current address: Department of Biology, Rhode Island College, Providence, RI 02908

**Keywords:** cilia, regeneration, *C. elegans*, DAF-19 RFX, intraflagellar transport, glia

## Abstract

Primary cilia are microtubule-based organelles essential for transducing environmental signals. While cilia reassemble upon cell cycle exit in dividing cells, it remains largely unknown whether postmitotic cells such as neurons can regenerate these structures *in vivo* following injury to restore function, and whether this process recapitulates developmental ciliogenesis. Here we show that a subset of sensory neuron cilia in adult *C. elegans* can regrow and restore neuronal functions following conditional truncation. This regeneration is regulated by both cell-intrinsic and extrinsic mechanisms that are in part distinct from those employed during embryonic ciliogenesis. We find that the conserved ciliogenic DAF-19 RFX transcription factor is dispensable for cilia maintenance in the adult but is essential for regrowth, in part via transcriptional upregulation of a subset of ciliary intraflagellar transport (IFT) genes. We also identify the DLK-1 dual leucine-zipper kinase and the CEBP-1 C/EBP transcription factor previously implicated in axon regeneration as necessary for efficient cilia regrowth but not for ciliogenesis. Finally, we show that cilia truncation and regeneration dynamics vary in a neuron type-specific manner and are modulated by signals from surrounding glia. Our results establish that the cilia of mature neurons can regenerate and recover functions *in vivo* and identify conserved pathways that regulate this process in adult animals.

## INTRODUCTION

Primary cilia are microtubule-based organelles that project from the membranes of most cell types and transduce environmental signals to regulate cellular and organismal functions ^1,2^. Consequently, loss of cilia or disruption of ciliary signaling underlies a broad class of disorders termed ciliopathies ^3,4^. Given the physical location of cilia on the cell surface, these organelles are particularly vulnerable to damage by chemical or physical agents ^5–8^. Cilia on airway epithelial cells can be injured by airborne pollutants, cilia on endothelial cells are disassembled in response to high laminar shear stress, and cilia on diverse cell types can be damaged upon viral infection ^5,7,9–12^. In dividing cells, damaged cilia can be replaced by highly regulated cycles of ciliary disassembly and reassembly correlated with cell-cycle entry and exit, respectively ^13,14^. However, whether cilia loss results in irreversible cellular dysfunction in postmitotic cells such as neurons, and whether cilia of different neuron types can regenerate and restore cellular function in the adult *in vivo* remain to be fully understood.

Cilia regeneration following injury or truncation has been described in multiciliated tissues, cultured cells, olfactory epithelia, the mammalian inner ear, and in unicellular organisms such as *Chlamydomonas* and *Tetrahymena* ^15–23^. In the mammalian brain, neuronal ciliogenesis occurs during embryonic and early postnatal development and cilia are then maintained through adult stages ^2,24–27^. Thus, if cilia were to regenerate following loss at later postnatal stages, regrowth would occur in neurons with developmental states and in cellular environments that differ significantly from those during embryonic development. These distinct internal and external conditions may preclude cilia regeneration in mature neurons or require mechanisms that differ from those driving developmental ciliogenesis. Analogous studies on axon regeneration across species have shown that axon regrowth in the adult relies on mechanisms that are partly distinct from those used for axon outgrowth and guidance during development ^28–31^. Moreover, while peripheral axons in mammals regenerate robustly, central axons exhibit limited regeneration due to both intrinsic and extrinsic factors ^32–35^.

In *C. elegans*, the majority of ciliated sensory neurons including the twelve neuron pairs of the bilateral head amphid organs are born in the embryo ^36^. Ciliogenesis in amphid neurons occurs during embryogenesis and these cilia are maintained throughout the life of these animals ^37–39^. Previous studies have shown that restoring ciliary gene function in the adult in ciliogenesis mutants can rescue cilia morphology, indicating that a subset of sensory cilia retains the capacity for regrowth in the adult ^39–41^. However, the intrinsic and extrinsic mechanisms that promote or inhibit cilia regeneration, and whether morphologically diverse cilia of different neuron types can regenerate in the adult, are unknown.

Here we show that a subset of amphid sensory neuron cilia formed during embryonic development can regenerate and restore neuronal functions in adult *C. elegans* following conditional truncation. We find that the essential and conserved ciliogenic DAF-19 RFX transcription factor ^42^ is dispensable for cilia maintenance in the adult but is required for regeneration, in part via upregulating intraflagellar transport (IFT) genes necessary for building cilia ^43,44^. We further identify roles for the DLK-1 dual leucine-zipper kinase and CEBP-1 C/EBP transcription factor, previously implicated in axon regeneration ^45,46^, in promoting cilia regrowth but not embryonic ciliogenesis. Cilia shorten and regrow with neuron-specific dynamics and we show that glial signals differentially regulate these processes across neuron types. Together, our results describe a set of cell-intrinsic and extrinsic mechanisms that regulate cilia regeneration in mature sensory neurons, and suggest that manipulation of these pathways may restore cilia and neuronal functions following injury or loss in adult animals.

## RESULTS

### Sensory cilia regenerate and restore neuronal function following conditional truncation in adult *C. elegans*

Pioneering studies on axon regeneration in adult *C. elegans* used laser microsurgery to sever individual axons and follow axon regrowth ^47,48^. The cilia of eight sensory neurons in the bilateral head amphid sense organs of *C. elegans* are ~5–7 μm long and are tightly bundled within a channel formed by glial processes (“channel” cilia) ^49,50^, making it impractical to perform high-throughput laser severing of individual cilia. *unc-70* β-spectrin gene mutants in which axons break spontaneously due to defects in the underlying neuronal membrane cytoskeleton have been used to increase throughput of genetic studies on axon regeneration ^51,52^. We, therefore, sought a genetic strategy to conditionally truncate cilia in adult *C. elegans* to initiate studies on cilia regeneration.

**Figure 1.**
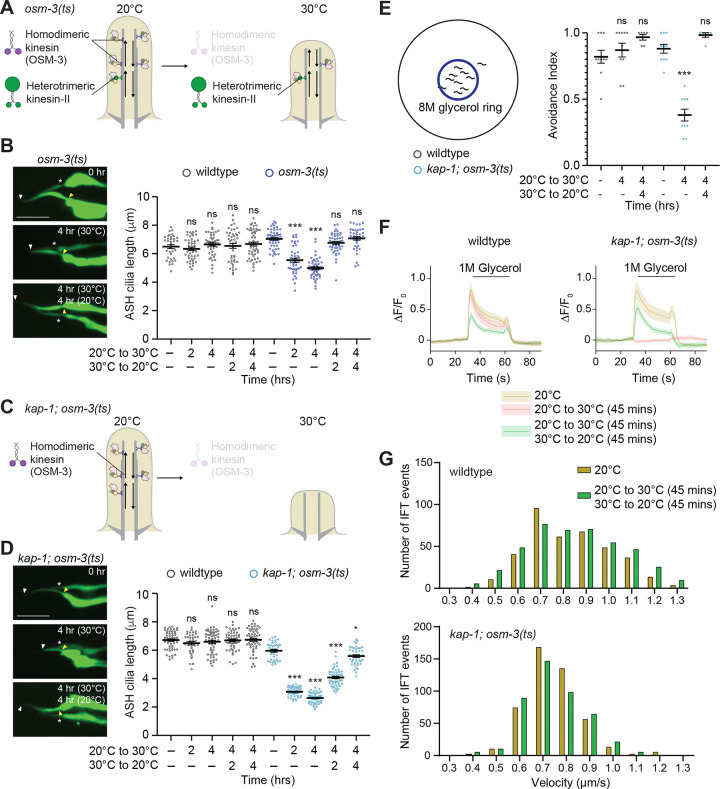
ASH cilia functionally regenerate upon truncation in adult *C. elegans.* **A,C)** Cartoons of ASH cilia in *osm-3(oy156ts)* (A) and *kap-1(ok676); osm-3(oy156ts)* (C) mutants before (left) and after (right) shift from the permissive (20°C) to the restrictive (30°C) temperature for 4+ hrs. Arrows indicate anterograde and retrograde IFT movement along the axoneme. **B,D)** (Left) Representative images of ASH cilia in *osm-3(oy156ts)* (B) and *kap-1(ok676); osm-3(oy156ts)* (D) mutants expressing *gfp* under the *sra-6* promoter at the indicated temperature shift conditions. Yellow/white arrowheads: cilia base/cilia tip; white asterisk: neighboring ASI cilium. Scale bar: 5 μm. (Right) Quantification of ASH cilia length in wildtype (black) and indicated mutants (blue) at the indicated temperature shift conditions. Each circle is the length of a single ASH cilium. ***: different at *P*<0.001 from unshifted within each genotype; ns: not significant (one-way ANOVA with Tukey’s multiple comparisons test). Errors are SEM. n≥36 each; 3 independent experiments. **E)** (Left) Cartoon of 8M glycerol avoidance assay. (Right) Quantification of 8M glycerol avoidance for wildtype (black) and *kap-1(ok676); osm-3(oy156ts)* mutants (blue) at the indicated temperature shift conditions. 0 and 1.0 indicate 0% and 100% avoidance, respectively. Each circle is a single assay of 10 animals. ***: different at *P*<0.001 from unshifted within each genotype; ns: not significant (one-way ANOVA with Tukey’s multiple comparisons test). Errors are SEM. n≥6 assays; 5 independent experiments. **F)** Mean GCaMP3 fluorescence changes in ASH to a 30s pulse of 1M glycerol in wildtype (left) and *kap-1(ok676); osm-3(oy156ts)* mutants (right) in the indicated temperature shift conditions. Shaded regions are SEM. n≥15 neurons each; 4 independent experiments. **G)** Histogram of velocities of reconstituted endogenously tagged OSM-6::split-GFP in ASH cilia in wildtype (top) and *kap-1(ok676); osm-3(oy156ts)* (bottom) animals. n≥19 animals per genotype; 3 independent experiments.

Cilia are built by the conserved process of IFT mediated by anterograde kinesin and retrograde dynein motors that traffic ciliary building blocks and signaling proteins into and out of these organelles ^43,44^. The middle segments of channel cilia in *C. elegans* are built by the partly redundant functions of a heterotrimeric kinesin-II and the homodimeric OSM-3 kinesin motor, whereas the distal segments are assembled by OSM-3 alone ^53,54^ (Figure 1A). We previously described the *osm-3(oy156)* temperature-sensitive (*ts*) allele which results in rapid truncation of channel cilia distal segments when adult *C. elegans* are shifted to the restrictive temperature of 30°C ^55^ (Figure 1A). We used this *ts* allele to test whether cilia can regenerate in the adult following truncation.

We focused our analysis primarily on the monociliated ASH nociceptive sensory neurons as a representative of channel cilia. Cilia were visualized via expression of GFP under the *sra-6* promoter which drives expression strongly in ASH and more weakly in the ASI monociliated channel chemosensory neurons ^49,50,56^ (see Materials and Methods). Consistent with previous observations ^55^, shifting *osm-3(ts)* adult animals to 30°C resulted in loss of the distal segments of the rod-like cilia of ASH neurons (Figure 1B). Truncation was gradual and was complete by 4 hrs (Figure 1B). When animals were returned to the permissive temperature of 20°C, the ASH cilia regrew to their original length within 2 hrs (Figure 1B). Cilia length in wildtype ASH neurons was unaffected by these temperature shifts (Figure 1B). This recovery of cilia morphology was more rapid than that observed previously using the *che-3(nx159ts)* allele in the tail phasmid organ sensory neurons ^40^.

While only distal segments are truncated in *osm-3* mutants, both middle and distal segments are lost in *kap-1; osm-3* loss-of-function double mutants that lack both anterograde IFT motors ^53,54^ (Figure 1C). To determine whether both middle and distal segments can regrow, we performed similar temperature shift experiments in *kap-1; osm-3(ts)* animals. ASH cilia were shorter by 1 hr, and severely truncated by 4 hrs, after the temperature upshift (Figure 1D, Figure S1). Upon returning to the permissive temperature, significant elongation of these cilia was observed within 1 hr and these cilia regrew to nearly their original length by 4 hrs (Figure 1D, Figure S1). We infer that both the middle and distal segments of ASH cilia regrow rapidly following truncation in adult *C. elegans*.

We next assessed whether sensory functions are restored in neurons with regrown cilia. ASH detects and drives avoidance of high osmolarity ^57^. Wildtype animals placed within a ring of 8M glycerol remain within the ring since they avoid this chemical. Animals lacking ASH cilia rapidly escape the ring since they fail to avoid high osmolarity (*osm* or osmotic avoidance defective) ^58^. At 20°C, *kap-1; osm-3(ts)* mutants avoided glycerol (Figure 1E). However, upon shifting to 30°C, these animals escaped the ring as ASH cilia were truncated (Figure 1E). Glycerol avoidance was fully restored after returning to 20°C correlating with cilia regrowth (Figure 1E). Consistently, glycerol evoked a robust increase in intracellular calcium in ASH neurons in *kap-1; osm-3(ts)* double mutants at the permissive temperature, but this response was abolished at the restrictive temperature (Figure 1F). Calcium responses were restored upon return to the permissive temperature (Figure 1F). Calcium dynamics were unaltered in wildtype animals at any examined temperature condition (Figure 1F). We conclude that recovery of cilia length restores neuronal functions.

We also determined whether IFT dynamics are restored upon cilia regrowth by analyzing the movement of the IFT-B particle component OSM-6/IFT52. To visualize IFT in ASH we used the previously described *osm-6(oy166)* allele in which *gfp*_*11*_ sequences are inserted at the endogenous *osm-6* locus via gene editing ^55^. GFP was reconstituted in ASH via expression of the GFP_1–10_ fragment under the *sra-6* promoter ^55,56^. Movement of OSM-6::split-GFP in the regrown ASH cilia was comparable to that observed in *kap-1; osm-3(ts)* animals at the permissive temperature (Figure 1G). Together, these results indicate that both sensory functions and IFT are fully restored in regrown ASH cilia. Henceforth we refer to these structures as regenerated cilia.

### The DAF-19 RFX transcription factor is necessary for cilia regeneration but not maintenance in the adult

The conserved DAF-19 RFX transcription factor is the master ciliogenic gene in *C. elegans* ^42^. This protein regulates the expression of all core ciliary genes including IFT genes by directly binding X box motifs in their upstream regulatory regions ^59–61^. Consequently, cilia fail to form in *daf-19* mutants ^42^. Although transcriptomic studies have suggested that DAF-19 is partly dispensable for cilia maintenance in later postembryonic stages ^62^, a role for this transcription factor in maintaining and regenerating cilia in the adult has not been tested.

**Figure 2.**
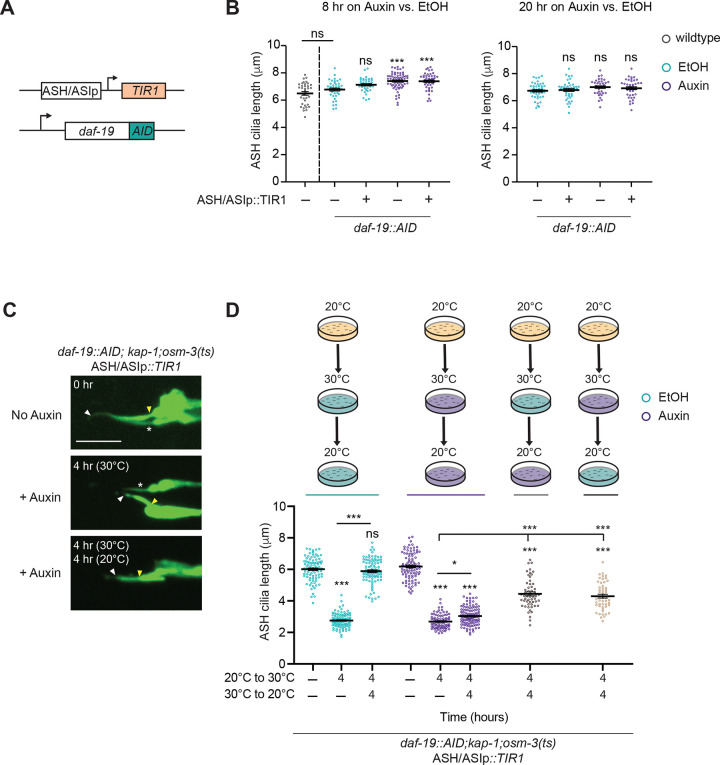
DAF-19 is required for cilia regeneration but not cilia maintenance. **A)** Schematic of endogenously tagged *daf-19::AID* together with TIR1 expressed under the *sra-6* promoter from an extrachromosomal array. The *sra-6* promoter used drives expression in ASH and ASI in the head ^56^. **B)** Quantification of ASH cilia length in animals containing *daf-19::AID* with or without the *sra-6*p*::TIR1* transgene on ethanol control (teal circles) or auxin-containing (purple circles) plates for 8 (left) or 20 (right) hrs. ASH cilia length in wildtype animals (black circles) is shown for comparison on the left (data are repeated from Figure 1B). Each circle is the length of a single ASH cilium. ***: different from ethanol control without TIR1 expression at P<0.001; ns: not significant (one-way ANOVA with Tukey’s multiple comparisons test). n≥37 each; 3 independent experiments. **C)** Representative images of ASH cilia expressing GFP in *daf-19(AID); kap-1(ok676); osm-3(oy156ts)* mutants together with *sra-6*p*::TIR1* in the indicated temperature shift conditions with or without auxin. Yellow/white arrowheads: cilia base/cilia tip; white asterisk: neighboring ASI cilium. Scale bar: 5 μm. **D)** (Top) Schematic of experimental conditions associated with cilia length quantifications (bottom). Each circle is the length of a single ASH cilium. * and **: different at *P*<0.05 and 0.001, respectively from unshifted within each condition or between indicated; ns: not significant (one-way ANOVA with Tukey multiple comparison’s test). Errors are SEM. n≥50 each; 6 independent experiments for continuous auxin or EtOH treatment, and 3 independent experiments for auxin treatment only during truncation or regrowth.

To test whether DAF-19 is required for cilia regeneration, we conditionally depleted DAF-19 specifically in the adult. We generated an endogenously tagged *daf-19* allele fused to an auxin-inducible degron (AID) ^63,64^ (Figure 2A). Animals carrying this allele did not exhibit cilia defects as assessed via their normal cilia lengths and ability to uptake a lipophilic dye (Figure 2B, Figure S2A), indicating that this allele retains function. We first determined whether acute depletion of DAF-19 in the adult disrupts cilia. Adult animals expressing *daf-19::AID* with or without the auxin receptor TIR1 expressed under the *sra-6* promoter in ASH/ASI (Figure 2A) were transferred to plates containing either the ethanol control or 4 mM auxin, and cilia morphology was assessed after 8 or 20 hrs (Figure 2B). ASH cilia length was largely unaffected under all examined conditions (Figure 2B). To confirm effective depletion, we examined GFP levels in animals expressing an endogenous functional *daf-19::AID::gfp* allele (Figure S2A) together with TIR1 expressed in ASH/ASI on plates containing auxin. GFP levels in both ASH and ASI appeared to be markedly reduced by 30 mins and were undetectable by 4 hrs of auxin exposure (Figure S2B). We conclude that DAF-19 is not necessary to maintain cilia in the adult.

We next determined whether DAF-19 is necessary for cilia truncation and/or regeneration by shifting adult *kap-1; osm-3(ts)* mutants carrying the *daf-19::AID* allele and expressing TIR1 in ASH/ASI to either the restrictive or permissive temperature in the continuous presence of ethanol or auxin and then assessing cilia length. Cilia truncated robustly upon a shift to 30°C in both conditions (Figure 2C, 2D, Figure S2C). However, while animals on ethanol regenerated their cilia upon return to 20°C, cilia failed to elongate in animals continuously exposed to auxin, indicating a requirement for DAF-19 in cilia regrowth (Figure 2C, 2D). ASH cilia also exhibited significant regrowth defects in animals placed on auxin only during the upshift to 30°C or only during the downshift to 20°C, although we observed partial regrowth under both conditions (Figure 2D). We note that DAF-19::GFP levels were not fully restored and cilia did not fully regenerate even after 20 hrs following auxin removal (Figure S2B, S2C) indicating that we were unable to deplete DAF-19 specifically during cilia truncation. Together, these results suggest that while DAF-19 is dispensable for maintenance of channel cilia in the adult, this transcription factor is essential for cilia regrowth, and may function both during cilia truncation and regeneration phases to promote efficient regeneration.

### The ciliogenic DAF-19d isoform regulates cilia regeneration

**Figure 3.**
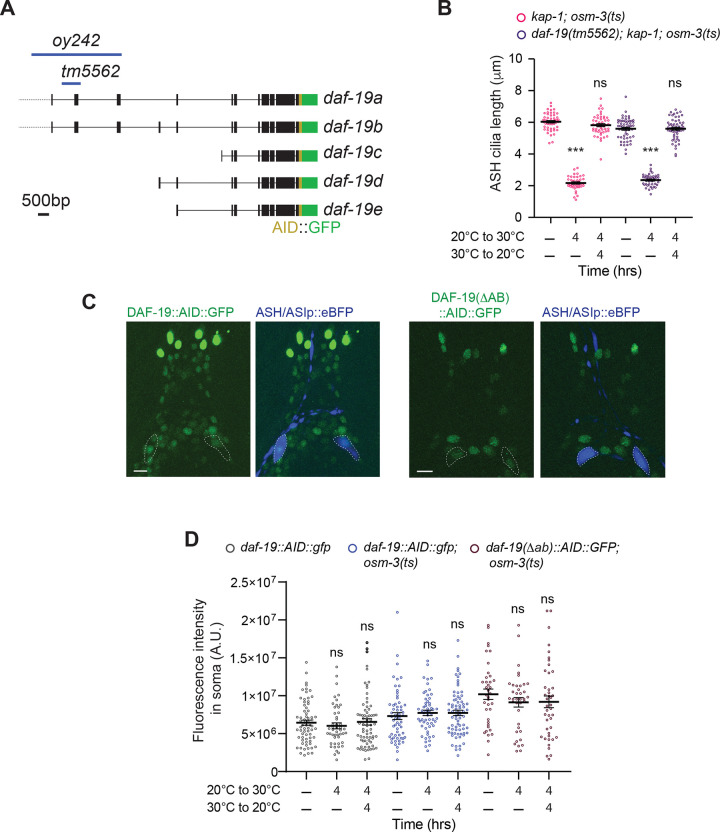
The ciliogenic *daf-19d* isoform regulates cilia regeneration. **A)** Schematic showing predicted *daf-19* isoforms (from www.wormbase.org) endogenously tagged with *AID::gfp* sequences. The molecular lesions in *daf-19(tm5562)* and *daf-19(oy242)* are indicated. **B)** Quantification of ASH cilia length in *kap-1; osm-3(ts)* or *daf-19(tm5562); kap-1; osm-3(ts)* mutants at the indicated temperature shift conditions. Each circle is the length of a single ASH cilium. ***: different at *P*<0.001 from unshifted within each genotype; ns: not significant (one-way ANOVA with Tukey multiple comparison’s test). Errors are SEM. n≥40 each; 3 independent experiments. **C)** Representative images of reporter expression from endogenously tagged *daf-19* and *daf-19(oy242Δab)* alleles in ASH/ASI soma marked via expression of *sra-6*p*::eBFP*. Anterior at top. Scale bars: 5 μm. **D)** Quantification of reporter fluorescence intensities in ASH/ASI soma of endogenously reporter tagged *daf-19* in wildtype (black) or *osm-3(oy156ts)* (blue), and endogenously reporter-tagged *daf-19(oy242ΔAB)* in *osm-3(oy156ts)* (brown) in the shown temperature shift conditions. Each circle is the combined value from ASH/ASI soma in one amphid organ. ns: not significant from unshifted within each genotype (one-way ANOVA with Tukey multiple comparison’s test). Errors are SEM. n≥38 each; 2 independent experiments.

The *daf-19* locus encodes multiple protein isoforms which share common C-terminal sequences and differ at their N-termini ^62,65–67^ (Figure 3A). The *daf-19d* isoform (previously *daf-19c*) has been specifically implicated in ciliogenesis ^62,65^. Since we were unable to selectively mutate *daf-19d* without also affecting other isoforms, we instead examined animals carrying the *daf-19(tm5562)* allele to determine whether *daf-19d* regulates cilia regeneration. The *daf-19(tm5562)* mutation disrupts the longer *daf-19a* and *daf-19b* isoforms and these mutants exhibit phenotypes consistent with loss of these isoforms while retaining *daf-19d* function ^62^ (Figure 3A). We observed no defects in either cilia truncation or regrowth in *daf-19(tm5562); kap-1; osm-3(ts)* triple mutants (Figure 3B), supporting the conclusion that *daf-19d* and not *daf-19a/b* regulates cilia regeneration.

We next examined whether DAF-19 protein levels are altered during cilia truncation and regeneration in adults. We did not detect altered expression of an endogenous reporter-tagged DAF-19 protein (Figure 3A) in ASH/ASI soma regardless of cilia length (Figure 3C, 3D). Since this allele contains *gfp* sequences fused to the common C-terminus (Figure 3A), we reasoned that upregulation of *daf-19d* alone may be masked by other isoforms. To examine *daf-19d* levels, we generated the *daf-19(oy242)* allele designed to disrupt expression of *daf-19a* and *daf-19b* while retaining expression of *daf-19c* (previously *daf-19m*), *daf-19d* and *daf-19e* isoforms (Figure 3A). *daf-19c* expression is restricted to the IL2 sensory neurons in hermaphrodites ^67^, the function of *daf-19e* is unknown. *daf-19(oy242Δab)* mutants did not exhibit dye-filling defects indicating that this allele does not disrupt ciliogenesis (Figure S2A). Reporter expression driven from the *daf-19(oy242Δab)* allele was also unaltered upon cilia truncation and regrowth (Figure 3C, 3D). These observations suggest that although DAF-19d is required for cilia regeneration and upregulation of IFT genes, its activity during this process is likely regulated by mechanisms other than increased expression (see Discussion).

### DAF-19 regulates distinct temporal dynamics of IFT gene expression during cilia truncation and regrowth

Complete regrowth of *Chlamydomonas* flagella following severing requires transcriptional upregulation of IFT and flagellar building block genes ^68–74^. We asked whether IFT genes are also upregulated in sensory neurons with regenerating cilia, and whether this upregulation is mediated by DAF-19.

**Figure 4.**
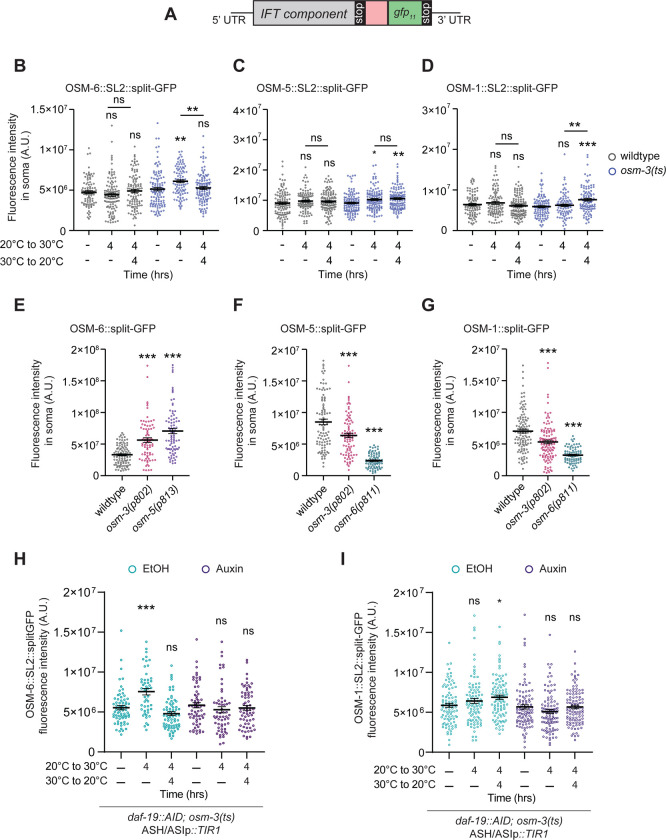
DAF-19 regulates temporal expression dynamics of IFT genes during cilia truncation and regeneration. **A)** Schematic of endogenously tagged transcriptional reporter. **B-D)** Quantification of fluorescence intensities in ASH/ASI soma of wildtype (black) or *osm-3(oy156ts)* (blue) animals expressing the indicated IFT transcriptional reporters in the shown temperature shift conditions. Each circle is the combined value from ASH/ASI soma in one amphid organ. *, ** and ***: different at *P*<0.05, 0.01 and 0.001, respectively, from unshifted within each genotype or between indicated; ns: not significant (one-way ANOVA with Tukey multiple comparison’s test). Errors are SEM. n≥85 each; 3 independent experiments. **E-G)** Quantification of fluorescence intensities of the indicated reporter-tagged IFT proteins in the ASH/ASI soma of wildtype or IFT gene mutants. Each circle is the combined value from ASH/ASI soma in one amphid organ. ***: different at *P*< 0.001 from wildtype (one-way ANOVA with Tukey’s multiple comparison test). Errors are SEM. n≥74 each; 3 independent experiments. **H,I)** Quantification of endogenously tagged OSM-6::SL2::split-GFP (H) or OSM-1::SL2::split-GFP (I) fluorescence intensities in ASH/ASI soma of *daf-19(AID); osm-3(ts)* mutants expressing *sra-6*p*::TIR1* in the indicated conditions. Each circle is the combined value from ASH/ASI soma in one amphid organ. *, ** and ***: different at *P*<0.05, 0.01, and 0.001, respectively from unshifted within each condition; ns: not significant (one-way ANOVA with Tukey’s multiple comparison test). Errors are SEM. n≥48 each; n>2 independent experiments.

To examine whether IFT gene expression is altered during cilia truncation or regrowth, we generated strains in which IFT genes are tagged at their endogenous loci with either *SL2::gfp*_*11*_ or *gfp*_*11*_ sequences alone, together with expression of *gfp*_*1–10*_ under the *sra-6* promoter to visualize reconstituted GFP selectively in ASH/ASI (Figure 4A, Figure S3A). Insertion of the *SL2* trans-splice leader sequence allows monitoring of transcriptional changes independent of protein localization. We examined the *osm-6/IFT52, osm-5/IFT88*, and *osm-1/IFT172* genes which encode conserved components of the IFT-B complex ^75,76^. Loss-of-function mutations in each of these genes result in severe ciliary structural defects ^77,78^.

Expression of *osm-6::SL2::split-GFP* was significantly upregulated in ASH/ASI in *osm-3(ts)* mutants when the distal segments were fully truncated, and subsequently returned to baseline levels as cilia regenerated (Figure 4B). OSM-6::split-GFP protein levels in ASH/ASI soma showed a similar pattern of upregulation although protein levels in cilia appeared unaltered (Figure S3B). In contrast, *osm-5::SL2::split-GFP* showed only weak upregulation upon cilia regeneration (Figure 4C), with no detectable changes in OSM-5 protein levels either in ASH/ASI soma or cilia (Figure S3C). *osm-1* exhibited distinct expression dynamics such that *osm-1* expression was significantly upregulated only upon cilia regrowth with the protein exhibiting similar expression changes in the ASH/ASI soma but not in cilia (Figure 4D, Figure S3D). Together, these results indicate that a subset of IFT genes is transcriptionally upregulated during cilia truncation and regeneration with distinct temporal patterns of expression.

We next asked whether altered IFT gene expression is correlated with cilium length and/or reflects a distinct cellular state associated with acute cilium disassembly or regeneration in the adult. To distinguish between these possibilities, we examined IFT gene expression in animals carrying loss-of-function mutations in the *osm-3* kinesin motor or other IFT genes which result in truncated cilia throughout development. As observed during acute conditional cilia truncation, OSM-6 protein levels and an *osm-6* transcriptional reporter were elevated in ASH/ASI soma in both *osm-3* and *osm-5* loss-of-function mutants (Figure 4E, Figure S3E). In contrast, levels of both OSM-5 and OSM-1 proteins were instead markedly reduced in animals carrying loss-of-function alleles of either *osm-3* or *osm-6* (Figure 4F, 4G). In all cases, expression changes were more pronounced in *osm-5* or *osm-6* mutants that exhibit severely truncated cilia than in *osm-3* mutants in which only the distal ciliary segments fail to assemble (Figure 4E–G, Figure S3E). These observations suggest that while OSM-6 levels may partly scale with cilia length regardless of condition, the expression of other IFT genes is regulated in a more complex manner by both cilia and developmental state.

We next tested whether DAF-19 is required for the upregulation of IFT genes during cilia truncation and regeneration. Consistent with the observation that DAF-19 is dispensable for cilia maintenance in the adult, expression levels of IFT genes were unaffected upon depletion of DAF-19 under control growth conditions (Figure 4H, 4I). However, the truncation and regeneration-associated upregulation of both *osm-6::SL2::gfp* and *osm-1::SL2::gfp* was abolished upon depletion of DAF-19 in the adult (Figure 4H, 4I). We conclude that while DAF-19 is not required to maintain IFT gene expression in the adult, this transcription factor is necessary for transcriptional upregulation of IFT gene expression likely supporting cilia regeneration.

### The DLK-1 MAPKKK and CEBP-1 C/EBP transcription factor promote cilia regeneration

The DLK dual leucine-zipper kinase is activated by disruption of the microtubule cytoskeleton upon axon injury and promotes axon regeneration via downstream transcriptional programs ^45,46,79–84^. Recent work in *C. elegans* has also shown that mutations in IFT genes increase DLK-1 accumulation in sensory cilia and that this kinase acts via the CEBP-1 C/EBP transcription factor to protect a subset of sensory cilia from degeneration and to regulate cilia length ^85,86^. Since the microtubule-based ciliary axoneme is disrupted during cilia truncation, we tested whether DLK-1 signaling contributes to efficient cilia regrowth.

**Figure 5.**
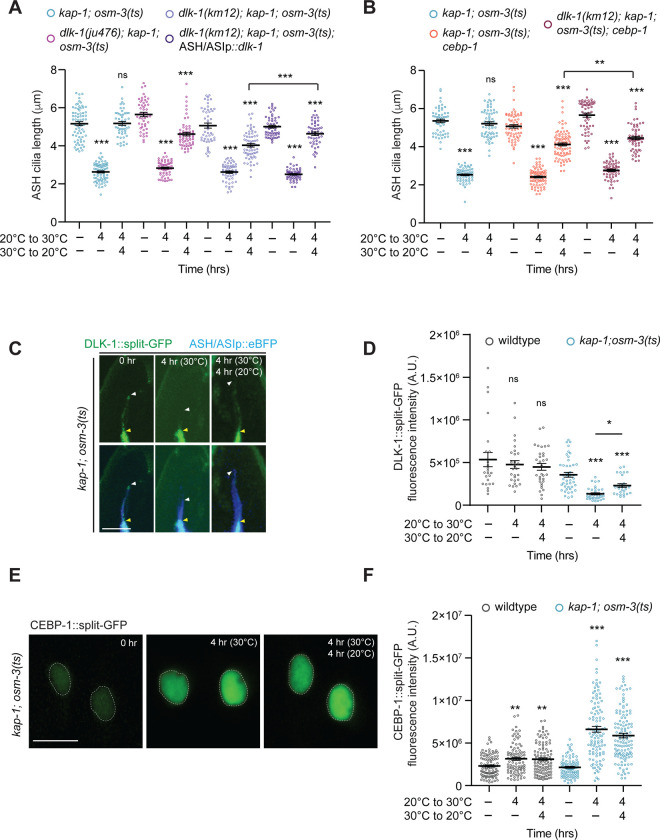
DLK-1 and CEBP-1 promote cilia regeneration. **A,B)** Quantification of ASH cilia length in the shown genetic backgrounds at the indicated temperature shift conditions. Wildtype *dlk-1* sequences were expressed in ASH/ASI under the *sra-6* promoter. Each circle is the length of a single ASH cilium. **, ***: different at *P*<0.01 and 0.001, respectively, from unshifted within each genotype or between indicated; ns: not significant (one-way ANOVA with Tukey’s multiple comparison test). Errors are SEM. n≥48 each; >3 independent experiments. **C,E)** Representative images of endogenously tagged DLK-1:split-GFP expression in ASH cilia (C) and CEBP-1::split-GFP in ASH/ASI soma (E) at the indicated temperature shift conditions in a *kap-1; osm-3(ts)* background. Yellow/white arrowheads: cilia base/cilia tip (C). Anterior is at top in (C). Scale bars: 5 μm. **D,F)** Quantification of fluorescence intensities of DLK-1::split-GFP in ASH cilia (D) and CEBP-1::split-GFP in ASH/ASI soma (F) in the indicated genetic backgrounds and temperature shift conditions. Each circle is the value from a single ASH cilium (D) or the combined value from ASH/ASI soma in one amphid organ (F). *, ** and ***: different at *P*<0.05, 0.01 and 0.001, respectively, from unshifted within each genotype or between indicated; ns: not significant (one-way ANOVA with Tukey’s multiple comparison test). Errors are SEM. n≥24 each (D), n>92 each (F); 3 independent experiments.

Loss-of-function mutations in *dlk-1* caused only mild defects in ASH cilia length (Figure S4A). We also did not observe effects on ASH cilia truncation in *dlk-1; kap-1; osm-3(ts)* triple mutants upon an upshift to 30°C (Figure 5A). However, upon return to the permissive temperature of 20°C, loss of *dlk-1* resulted in a failure of cilia to fully regenerate to their original length by 4 hrs (Figure 5A). This defect was rescued upon expression of wildtype *dlk-1* sequences in ASH/ASI (Figure 5A). The partial regeneration of cilia in *dlk-1* mutants after 4 hrs at the permissive temperature suggested that loss of *dlk-1* may delay but not fully abolish cilia regeneration. Consistent with this notion, cilia in *dlk-1* mutants eventually regenerated to the wildtype length after 16 hrs at the permissive temperature (Figure S4B). Previous work reported that movement of the kinesin-II motor is slowed in ciliary middle segments in *dlk-1* mutants ^86^. However, we did not detect changes in IFT-mediated transport of endogenously tagged OSM-6 in *dlk-1* mutants (Figure S4C), suggesting that slower IFT dynamics may not be the primary cause of delayed regrowth in *dlk-1* mutants. We infer that DLK-1 is required for efficient cilia regeneration.

DLK-1 regulates axon regeneration through well-characterized MAP kinase signaling cascades (Figure S4D) ^29^. To determine whether these pathways contribute to cilia regeneration, we examined mutants in downstream signaling components. Mutations in *mlk-1* MAPKKK or *pmk-3* MAPK mutants did not affect cilia regrowth, although loss of *kgb-1* JNK-like MAPK caused a significant defect in cilia regeneration (Figure S4E). We next examined whether the downstream transcription factor CEBP-1 is required for cilia regeneration. As observed for *dlk-1* mutants, loss of *cebp-1* had only a minor effect on ASH cilia length (Figure S4A) but affected ASH cilia regeneration in the *kap-1; osm-3(ts)* mutant background (Figure 5B). This regeneration defect was altered to only a minor albeit significant extent in *dlk-1; cebp-1* double mutants suggesting that these molecules act in a linear pathway to promote cilia regeneration (Figure 5B).

DLK-1 levels are low in cilia in wildtype animals but are increased upon cilia truncation in IFT mutants ^85^. Similarly, CEBP-1 levels increase in the soma of sensory neurons in IFT mutants ^85^. Endogenously tagged DLK-1::split-GFP ^85^ was present at low levels in ASH/ASI cilia in both wild-type animals and in *kap-1; osm-3(ts)* mutants at 20°C (Figure 5C, 5D). DLK-1 levels were further decreased in these cilia upon truncation in the double mutant, but increased significantly upon cilia regrowth (Figure 5C,D). We also examined expression of a nuclear-localized endogenously tagged CEBP-1::split-GFP reporter ^87^. Levels of this protein in ASH/ASI soma were significantly increased in *kap-1; osm-3(ts)* mutants upon truncation and remained elevated when cilia were fully regrown (Figure 5E, 5F). Together, these results support the hypothesis that DLK-1 and CEBP-1 act in a linear signaling pathway to promote efficient cilia regeneration.

### Glial signals differentially influence neuron type-specific dynamics of cilia truncation and regeneration

**Figure 6.**
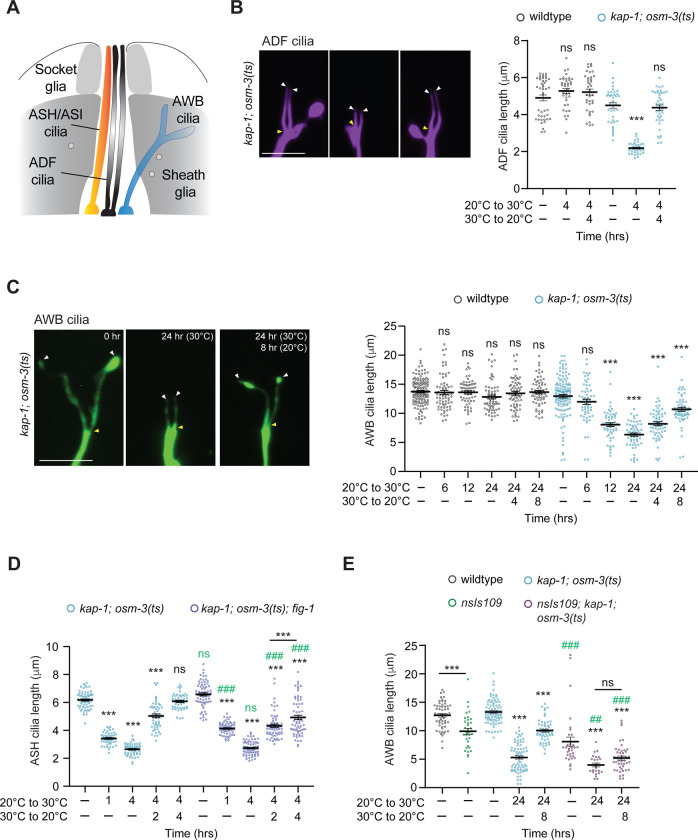
Glia modulate cilia regeneration dynamics in a cell type-specific manner. **A)** Schematic of the cilia of the ASH/ASI, ADF, and AWB neurons with associated sheath and socket glia in the amphid sense organ of the head. Anterior is at top. Adapted from ^77^. **B,C)** Representative images (left) and quantification of ADF (B) and AWB (D) cilia lengths (right) in the shown genetic backgrounds at the indicated temperature shift conditions. Anterior at top. Scale bar: 5 μm. Each circle is the average length of both ADF or AWB cilia from a single neuron. ***: different at *P*<0.001 from unshifted within each genotype; ns: not significant (one-way ANOVA with Tukey’s multiple comparison test). Errors are SEM. n≥38 each; >3 independent experiments. **D,E)** Quantification of ASH (D) and AWB (E) cilia length in the shown genetic backgrounds at the indicated temperature conditions. Each circle is the length of a single ASH cilium (D) or the average length of both AWB cilia from a single neuron (E). ***: different at *P*<0.001 from unshifted within each genotype or between indicated; ns: not significant (one-way ANOVA with Tukey’s multiple comparison test). ###: different at *P*<0.001 from the *kap-1; osm-3(ts)* genotype at the corresponding timepoint; ns (green): not significant (t-test). Errors are SEM. n≥25 each; >3 independent experiments.

Sensory cilia in *C. elegans* exhibit remarkably diverse morphologies ^49,50,77^. We next tested whether distinct cilia types can undergo truncation and regeneration in the adult. Unlike the monociliated ASH or ASI neurons, the ADF neurons contain two cilia that are also present in the amphid channel (Figure 6A, 6B) ^49,50,77^. In *kap-1; osm-3(ts)* mutants, these cilia truncated and regrew with dynamics similar to those observed for ASH cilia following shifts to the restrictive and permissive temperatures, respectively (Figure 6B). The AWB olfactory neurons contain morphologically complex cilia that are embedded in the amphid sheath glial processes unlike channel cilia (Figure 6A, 6C) ^49,50,77^. In *kap-1; osm-3(ts)* mutants, AWB cilia truncated and regenerated, but did so on significantly longer timescales than ASH cilia. AWB cilia were only partly truncated even after 24 hrs at 30°C (average AWB cilium length in *kap-1; osm-3(lof)* and *kap-1; osm-3(ts)* double mutants is 2.9 ± 0.2 μm and 6.4 ± 2.1 μm, respectively), and these cilia failed to regrow to their original length even after 8 hrs at 20°C (Figure 6C). We infer that diverse sensory cilia types in adult *C. elegans* are able to truncate and regrow, but that the dynamics of these processes vary in a neuron type-specific manner.

While mammalian peripheral axons regenerate robustly following axotomy, central axons fail to do so efficiently, in part due to inhibitory signals from glia and the extracellular matrix ^32,33^. In *C. elegans*, the amphid sheath glia regulate cilia morphology and protect cilia from degeneration ^88–91^. Disruption of channel cilia results in accumulation of glia-secreted extracellular matrix into the amphid channel; this glial response is neuroprotective ^89^. We tested whether glia influence neuron-specific dynamics of cilia truncation and regeneration.

We ablated the sheath glia using a previously described strain in which diptheria toxin A is expressed specifically in the sheath glia following amphid development ^88^. We noted significant defects in ASH cilia morphology in the *kap-1; osm-3(ts)* double mutant strain in the glia-ablated background even at the permissive temperature that precluded further analyses of cilia truncation and regrowth in this strain (Figure S5A). Channel cilia integrity has been proposed to be monitored by interactions between the cilia-localized DGS-1 transmembrane and glia-expressed thrombospondin domain FIG-1 proteins ^89^. Mutations in either *dgs-1* or *fig-1* trigger the glial protective secretory response even in animals with intact channel cilia ^89^. Mutations in *fig-1* did not alter ASH cilia length at the permissive temperature (Figure 6D). However, in *kap-1; osm-3(ts); fig-1* mutants ASH cilia truncated more slowly upon a shift to 30°C, and failed recover to their original length after 4 hrs at the permissive temperature (Figure 6D). These observations suggest that the glial secretome modulates the dynamics of channel cilia truncation and regrowth.

Although AWB cilia structure was reported to be largely retained upon sheath glia ablation ^88^, we found that AWB cilia length and morphology were also significantly affected in these animals and further shortened in a *kap-1; osm-3(ts)* background even at the permissive temperature (Figure 6E, Figure S5B). AWB cilia exhibited additional truncation upon a temperature upshift and failed to regrow upon return to the permissive temperature (Figure 6E, Figure S5B). These observations further support a role for glia in modulating cilia truncation and regrowth, and suggest that these mechanisms operate in a neuron type-specific manner in the adult.

## DISCUSSION

Here we show that sensory neuron cilia in adult *C. elegans* can regenerate and restore neuronal function. We find that while the DAF-19 RFX ciliogenic transcription factor is not necessary for maintaining cilia in the adult, it is required for cilia regeneration in part via regulation of IFT gene expression during cilia truncation and regrowth. We also find that the DLK-1 kinase and CEBP-1 transcription factor, previously implicated in promotion of axon regeneration and protection against cilia degeneration, are necessary for efficient cilia regrowth. Finally, we show that cilia truncation and regeneration dynamics vary across neuron types and are modulated by glia. Our results establish that both cell-intrinsic and extrinsic mechanisms promote robust neuronal cilia regeneration in the adult, and that variations in these pathways contribute to neuron type-specific differences in cilia regenerative capacities.

*Chlamydomonas* flagella elongate to half their original length following truncation if new protein synthesis is inhibited indicating that pre-existing ciliary building blocks are sufficient to support partial flagella regrowth in the absence of increased flagellar gene expression ^68^. In contrast, we find that loss of *daf-19* abolishes cilia elongation in *C. elegans,* suggesting that DAF-19-dependent transcriptional changes are essential for cilia regeneration in this organism. As in *Chlamydomonas* ^72,92,93^, we observed distinct temporal patterns of ciliary gene expression during cilia truncation and regeneration in *C. elegans* such that *osm-6* but not other IFT genes is upregulated prior to initiation of cilia regrowth. Recent cryo-EM analyses have shown that OSM-6/IFT52 forms a central scaffold of the IFT-B1 complex ^75^. Early upregulation of the OSM-6 core component upon cilia truncation may promote the ordered assembly of pre-existing IFT complexes to support efficient cilia regeneration. Consistently, we also observed marked upregulation of OSM-6 but not other examined IFT genes, in IFT *lof* mutants. In the future, it will be important to identify the entire complement of genes whose expression is altered during cilia truncation and/or regrowth in a neuron type-specific manner, and to determine whether these transcriptional responses are mediated solely by DAF-19 or also by other factors across neuron types.

How might IFT gene expression be regulated on distinct timescales during cilia disassembly and regeneration? In neurons, activity-dependent and calcium-regulated gene expression occurs in distinct temporal waves driven in part via the early induction of transcription factors which subsequently regulate delayed expression of downstream target genes ^94–96^. Calcium has also been implicated in the regulation of flagellar gene expression and regeneration in *Chlamydomonas* ^97,98^. We speculate that cilia truncation triggers a similar transcriptional cascade with different factors cooperating with, or regulated by, DAF-19 to modulate ciliary gene expression on different timescales. Consistent with this notion, RFX proteins have been shown to act with transcription factors such as CREB and MEF2C to mediate activity- and calcium-regulated neuronal gene expression ^99–101^. DAF-19 functions may be similarly regulated by interactions with signaling-responsive factors potentially including CEBP-1, and/or by post-translational modifications, to dynamically shape ciliary gene expression during cilia truncation and regeneration ^102,103^.

An unexpected finding was that although DAF-19 is essential for the expression of all known ciliary genes ^42,59,61^, this protein is not required for maintenance of IFT gene expression in adult neurons. In *C. elegans*, the FKH-8 forkhead domain transcription factor acts synergistically with DAF-19 to regulate IFT gene expression, although *fkh-8* mutants exhibit relatively minor defects in amphid channel cilia morphology or IFT gene expression in these neurons ^104^. It is possible that DAF-19 cooperates with members of other transcription factor families to maintain ciliary gene expression in mature amphid sensory neurons ^105,106^. However, such factors might have been expected to have been identified from extensive forward genetic screens for ciliary defective mutants ^77,107,108^. Alternatively, ciliary gene loci may remain in an open chromatin state following ciliogenesis, and/or ciliary proteins may be highly stable in mature neurons, thereby reducing the requirement for continuous DAF-19-dependent transcription.

Disruption of the microtubule cytoskeleton is a key trigger for axon regeneration. Microtubule depolymerization activates DLK and downstream transcription factors independently of calcium signaling to promote axon regeneration ^81,83,84^, and recent work has shown that mutations in a β-tubulin subunit increase DLK-1 signaling ^109^. In the amphid organ, cilia disruption increases ciliary DLK-1 levels ^85,110^. In contrast, we observed reduced ciliary localization of DLK-1 in ASH/ASI upon cilia truncation. This discrepancy may arise from the use of different genetic mutants to truncate cilia or neuron type-specific patterns of DLK-1 localization. However, expression of *cebp-1* is increased in both contexts by as yet unknown mechanisms. Loss of DLK-1 slows but does not abolish cilia regeneration, indicating that this pathway promotes efficient cilia regeneration but is not essential for regrowth. This pathway may regulate cilia regeneration by modulating tubulin posttranslational modifications, inhibiting depolymerizing kinesins, or regulating endocytosis ^84,86,111^. Although we observe relatively minor effects on cilia length in *dlk-1* or *cebp-1* mutants under basal conditions, it remains possible that these molecules also contribute to efficient cilia elongation during embryogenesis.

The cilia of several sensory neurons in *C. elegans* are intimately associated with specific glia ^77,112^. Amphid sheath glia secrete components of the amphid channel matrix, engulf ciliary fragments and extracellular vesicles produced by sensory neurons, and monitor cilia integrity via direct interactions between glia and ciliary proteins such as FIG-1 and DGS-1, respectively ^88,89,91,113^. We find that disruption of glial-channel interactions slows both cilia truncation and regrowth in ASH, whereas glial loss abrogates cilia regrowth in AWB. There appear to be distinct glial microdomains associated with the glia-embedded distal sensory endings of individual neurons; these microdomains help maintain and shape cilia and sensory ending morphologies ^90,114–117^. Recent work has also shown that during aging, channel neurons protect glia-embedded neurons by inducing the unfolded protein response in amphid sheath glia ^118^, suggesting an intricate interaction among neurons and glia in maintaining cilia and thus neuronal functions in the adult.

Cilia regeneration in *C. elegans* exhibits intriguing parallels with axon regeneration mechanisms described in both worms and other organisms. In both processes, neuron-intrinsic pathways including DLK/CEBP1 signaling and induction of pro-regenerative gene expression, as well as extrinsic pathways such as glial cues contribute to structural and functional recovery in a neuron-specific manner ^29,30,33,35,119^. Moreover, axon regeneration is mediated by mechanisms that are partly distinct from those employed during developmental axon outgrowth. Similarly, our results suggest that cilia regrowth may also be driven in part by regeneration-specific programs. Further characterization of the pathways that promote or inhibit cilia regeneration may enable restoration of cilia function in postmitotic cells following injury thereby ameliorating phenotypes associated with acute cilia loss in the adult.

## MATERIALS and METHODS

### *C. elegans* genetics

*C. elegans* strains were cultured on nematode growth medium (NGM) plates seeded with *Escherichia coli* OP50. All strains were generated using standard genetic techniques. Mutations were verified through PCR-based amplification and/or DNA sequencing. Experimental plasmids were co-injected with either *unc-122*p::GFP or *unc-122*p::dsRed at a concentration of 40 ng/μl to establish transgenic lines. For each injected construct, animals from at least two independent transgenic lines were assessed, with one representative line analyzed in detail. The same extrachromosomal array was examined in both wildtype and mutant backgrounds for comparison. All experiments were conducted using one-day-old adult hermaphrodites unless specified otherwise.

### *C. elegans* genome-engineered strains

Endogenously tagged strains were generated using CRISPR/Cas9 genome engineering with Cas9 protein, tracrRNA, and crRNAs from IDT, as previously described ^55,120–122^. Repair templates included ssODNs or donor PCR products. All insertion sequences were added immediately before the stop codon (C-terminal tags) and confirmed by sequencing. The *dlk-1::gfp*_*11*_ allele was a gift from Yishi Jin ^85^. The *cebp-1::gfp*_*11*_ allele was a gift from Samuel Bates ^87^.

### Molecular Biology

The *dlk-1L* cDNA was a kind gift from Yishi Jin. The *sra-6*p*::dlk-1L*, *sra-6*p*::TIR1::SL2::mScarlet,* and *sra-6*p*::eBFP2* (gift from Nikhila Krishnan) plasmids were generated using standard cloning methods. All constructs were verified by sequencing.

### Osmotic avoidance assays

Osmotic avoidance assays were performed as previously described ^58^. Briefly, adult worms were first transferred to an NGM plate without food for 5 mins. 10 young adult hermaphrodites were then picked without food into the center of an 8M glycerol ring visualized with xylene cyanol (Sigma-Aldrich X4126). Animals were allowed to move freely on the plate, and after 10 mins the number of animals remaining within the ring were quantified.

### Calcium imaging

Stimulus-evoked imaging of intracellular calcium dynamics in ASH soma was performed using custom microfluidics devices as described previously ^55,123^. Images were acquired using an Olympus BX52WI microscope with a 40x oil objective and Hamamatsu Orca CCD camera at 4 Hz with 4 × 4 binning. Glycerol was diluted in filtered S-basal buffer, and wildtype and mutant worms were paralyzed in 10mM (−)-tetramisole hydrochloride (Sigma L9756) for several mins prior to loading into the microfluidics devices. The 1M glycerol stimulus was prepared fresh prior to each day of imaging, and mutant animals were imaged alongside wildtype controls in each independent experiment. Glycerol-evoked calcium transients in ASH were recorded for 2 cycles of 30 sec S-basal buffer/30 sec 1M glycerol/30 sec S-basal buffer. Data shown are recorded from the second stimulus pulse, as responses to the first pulse are variable. The 0 sec time point for glycerol responses represents the 90 sec following the start of calcium imaging.

Fluorescence changes were analyzed using a custom FIJI script ^55,124^ Images were first aligned using the Template Matching plugin, and manually drawn ROIs were used to identify soma and background regions. The background fluorescence was subtracted from that in the soma, and these values were used for further analysis in RStudio. RStudio was used to calculate the response mean and SEM, and F_0_ was calculated from the average of ΔF/F_0_ value for 5 sec prior to odor onset.

### Auxin experiments

4mM auxin containing plates were made by dissolving 400mM Auxin (1-naphthaleneacetic acid, Sigma 317918) in 100% ethanol and then adding to NGM. Control plates contained an equal volume of ethanol alone. Plates were seeded with 200 μl *E. coli* OP50, stored at 4 °C and wrapped in aluminum foil to prevent light exposure. For experiments in which cilia lengths were measured, animals were placed on auxin or control ethanol plates for at least 30 mins prior to temperature upshift or downshift. For experiments in which reporter fluorescence intensities were measured, animals were placed on auxin or control ethanol plates overnight prior to temperature upshift or downshift.

### Dye-filling

Stock solutions of 1,1′-dioctadecyl-3,3,3′,3′-tetramethylindocarbocyanine perchlorate (DiI) at 1 mg/ml in *N,N*-dimethylformamide (Sigma-Aldrich 468495) were stored at −20°C. To evaluate dye uptake, animals were immersed in 0.1mg/ml dye for one hour before examination.

### Fluorescent reporter imaging and image analysis

One day-old adult hermaphrodites were immobilized using 10mM tetramisole hydrochloride (Sigma-Aldrich L9756) and mounted on 10% agarose pads prepared on microscope slides with coverslips. Animals were imaged on an Andor CSU-W1 spinning disc confocal microscope with a Leica DM6000b body using a 100X objective. Images were acquired using Andor Fusion software version 2.4.0.14. Additional imaging was performed on a 3i Marianas spinning disc confocal microscope with a Zeiss Observer Z1 body equipped with a Yokagawa CSU-X1 spinning disk confocal head using a 100x objective. Images were acquired using Slidebook software. *z*-stacks were acquired with a step-size of 0.2 μm. For Figure 3C and Figure 5C, 5D, animals were imaged on an Andor Dragonfly 620SR spinning disc confocal microscope with a Nikon Ti2 body with an ASI Piezo Z-stage using a 60x objective. Images were acquired using Andor Fusion software version 2.7.0. *z*-stacks were acquired with a step-size of 0.2 μm.

Imaging parameters were kept consistent between wildtype and mutant samples. For cilia length measurements, image processing and analysis was conducted using FIJI/ImageJ (NIH). ASH cilia were identified as expressing *sra-6*p*::gfp* at higher levels than in neighboring ASI cilia. For fluorescent reporter intensity measurements, cell bodies or cilia were rendered in 3D by absolute intensity thresholding using the Surface tool in Imaris 10.2 (Oxford Instruments), prior to plotting sum intensity values. Since ASH and ASI neurons could not be differentiated by reporter expression levels, measurements included cilia or soma of both neurons.

### IFT Measurements

Intraflagellar transport (IFT) analyses were carried out as described previously ^55^. In brief, time-lapse recordings of fluorescently tagged IFT proteins in ASH cilia were carried out on a 3i Marianas spinning disc confocal microscope with a Zeiss Observer Z1 body equipped with a Yokagawa CSU-X1 spinning disk confocal head or an Andor CSU-W1 spinning disc confocal microscope with a Leica DM6000b body. Images were captured over a 30sec time frame at 250 msec exposure intervals and at 100X magnification. In FIJI/ImageJ, a line was manually drawn from the base through the middle segment of ASH cilia. Kymographs were generated using the Multi Kymograph and Template Matching plugins (ImageJ) to account for motion-related artifacts. After manually tracking IFT particles, velocity measurements were obtained by tracing line segments along each individual IFT trajectory.

### Statistical analyses

All graphs were generated and analyzed using RStudio (for calcium imaging analysis) and Prism 9 software. For comparisons among multiple strains, analyses used a one-way ANOVA followed by Tukey’s multiple comparisons test. The number of animals examined in each experiment, significance values, and number of independent experiments are provided in the figure legends.

## Supplementary Material

Supplement 1

## References

[1] Hilgendorf K.I., Myers B.R., and Reiter J.F. (2024). Emerging mechanistic understanding of cilia function in cellular signalling. Nat Rev Mol Cell Biol 7, 555–573. 10.1038/s41580-023-00698-5 PMC11199107 38366037

[2] Jurisch-Yaksi N., Wachten D., and Gopalakrishnan J. (2024). The neuronal cilium - a highly diverse and dynamic organelle involved in sensory detection and neuromodulation. Trends Neurosci 47, 383–394. 38580512 10.1016/j.tins.2024.03.004

[3] Reiter J.F., and Leroux M.R. (2017). Genes and molecular pathways underpinning ciliopathies. Nat Rev Mol Cell Biol 18, 533–547. 28698599 10.1038/nrm.2017.60 PMC5851292

[4] Andreu-Cervera A., Catala M., and Schneider-Maunoury S. (2021). Cilia, ciliopathies and hedgehog-related forebrain developmental disorders. Neurobiol Dis 150, 105236. 33383187 10.1016/j.nbd.2020.105236

[5] Iomini C., Tejada K., Mo W., Vaananen H., and Piperno G. (2004). Primary cilia of human endothelial cells disassemble under laminar shear stress. J Cell Biol 164, 811–817. 15024030 10.1083/jcb.200312133 PMC2172280

[6] Heller R.F., and Gordon R.E. (1986). Chronic effects of nitrogen dioxide on cilia in hamster bronchioles. Exp Lung Res 10, 137–152. 3956447 10.3109/01902148609061489

[7] Wang L., Liu C., Yang B., Zhang H., Jiao J., Zhang R., Liu S., Xiao S., Chen Y., Liu B., (2022). SARS-CoV-2 ORF10 impairs cilia by enhancing CUL2ZYG11B activity. J Cell Biol 221, e202108015. 35674692 10.1083/jcb.202108015 PMC9184850

[8] Lewin R.A., Lee T.H., and Fang L.S. (1982). Effects of various agents on flagellar activity, flagellar autotomy and cell viability in four species of Chlamydomonas (chlorophyta: volvocales). Symp Soc Exp Biol 35, 421–437. 6764045

[9] Butowt R., Bilinska K., and von Bartheld C.S. (2023). Olfactory dysfunction in COVID-19: new insights into the underlying mechanisms. Trends Neurosci 46, 75–90. 36470705 10.1016/j.tins.2022.11.003 PMC9666374

[10] Cao Y., Chen M., Dong D., Xie S., and Liu M. (2020). Environmental pollutants damage airway epithelial cell cilia: Implications for the prevention of obstructive lung diseases. Thorac Cancer 11, 505–510. 31975505 10.1111/1759-7714.13323 PMC7049516

[11] Gupta A., Thirugnanam K., Thamilarasan M., Mohieldin A.M., Zedan H.T., Prabhudesai S., Griffin M.R., Spearman A.D., Pan A., Palecek S.P., (2022). Cilia proteins are biomarkers of altered flow in the vasculature. JCI insight 7, e151813. 35143420 10.1172/jci.insight.151813 PMC8986075

[12] Horton K., Wing P.A.C., Jackson C.L., McCormick C.J., Carroll M.P., and Lucas J.S. (2025). Interplay between respiratory viruses and cilia in the airways. Eur Respir Rev 34, 240224. 40107662 10.1183/16000617.0224-2024 PMC11920889

[13] Cajanek L., Smite S., Ivashchenko O., and Huranova M. (2025). Cilia at the crossroad: convergence of regulatory mechanisms to govern cilia dynamics during cell signaling and the cell cycle. Cell Biosci 15, 81. 40483459 10.1186/s13578-025-01403-z PMC12144771

[14] Seeley E.S., and Nachury M.V. (2010). The perennial organelle: assembly and disassembly of the primary cilium. J Cell Sci 123, 511–518. 20144999 10.1242/jcs.061093 PMC2818191

[15] Lefebvre P.A., Nordstrom S.A., Moulder J.E., and Rosenbaum J.L. (1978). Flagellar elongation and shortening in *Chlamydomonas*. IV. Effects of flagellar detachment, regeneration, and resorption on the induction of flagellar protein synthesis. J Cell Biol 78, 8–27. 149796 10.1083/jcb.78.1.8 PMC2110168

[16] Werner M.E., and Mitchell B.J. (2013). Using *Xenopus* skin to study cilia development and function. Methods Enzymol 525, 191–217. 23522471 10.1016/B978-0-12-397944-5.00010-9 PMC4319646

[17] Overgaard C.E., Sanzone K.M., Spiczka K.S., Sheff D.R., Sandra A., and Yeaman C. (2009). Deciliation is associated with dramatic remodeling of epithelial cell junctions and surface domains. Mol Biol Cell 20, 102–113. 19005211 10.1091/mbc.E08-07-0741 PMC2613083

[18] Rosenbaum J.L., and Carlson K. (1969). Cilia regeneration in *Tetrahymena* and its inhibition by colchicine. J Cell Biol 40, 415–425. 4882889 10.1083/jcb.40.2.415 PMC2107625

[19] Rosenbaum J.L., and Child F.M. (1967). Flagellar regeneration in protozoan flagellates. J Cell Biol 34, 345–364. 6033540 10.1083/jcb.34.1.345 PMC2107224

[20] Cancalon P. (1983). Influence of a detergent on the catfish olfactory mucosa. Tissue Cell 15, 245–258. 6879593 10.1016/0040-8166(83)90020-4

[21] Rao V.G., Subramanianbalachandar V.A., Magaj M.M., Redemann S., and Kulkarni S.S. (2025). Mechanisms of cilia regeneration in *Xenopus* multiciliated epithelium in vivo. EMBO Rep 26, 2192–2220. 40087471 10.1038/s44319-025-00414-8 PMC12019409

[22] Forge A., Li L., Corwin J.T., and Nevill G. (1993). Ultrastructural evidence for hair cell regeneration in the mammalian inner ear. Science 259, 1616–1619. 8456284 10.1126/science.8456284

[23] Rannestad J. (1974). The regeneration of cilia in partially deciliated *Tetrahymena*. J Cell Biol 63, 1009–1017. 4215818 10.1083/jcb.63.3.1009 PMC2109370

[24] Arellano J.I., Guadiana S.M., Breunig J.J., Rakic P., and Sarkisian M.R. (2012). Development and distribution of neuronal cilia in mouse neocortex. J Comp Neurol 520, 848–873. 22020803 10.1002/cne.22793 PMC3325766

[25] Chen S., Alhassen W., Vakil Monfared R., Vachirakorntong B., Nauli S.M., Baldi P., and Alachkar A. (2021). Dynamic changes of brain cilia transcriptomes across the human lifespan. Int J Mol Sci 22, 10387. 34638726 10.3390/ijms221910387 PMC8509004

[26] Tereshko L., Turrigiano G.G., and Sengupta P. (2022). Primary cilia in the postnatal brain: Subcellular compartments for organizing neuromodulatory signaling. Curr Opin Neurobiol 74, 102533. 35405626 10.1016/j.conb.2022.102533 PMC9167775

[27] Guemez-Gamboa A., Coufal N.G., and Gleeson J.G. (2014). Primary cilia in the developing and mature brain. Neuron 82, 511–521. 24811376 10.1016/j.neuron.2014.04.024 PMC4104280

[28] He Z., and Jin Y. (2016). Intrinsic control of axon regeneration. Neuron 90, 437–451. 27151637 10.1016/j.neuron.2016.04.022

[29] Chisholm A.D., Hutter H., Jin Y., and Wadsworth W.G. (2016). The genetics of axon guidance and axon regeneration in *Caenorhabditis elegans*. Genetics 204, 849–882. 28114100 10.1534/genetics.115.186262 PMC5105865

[30] Hao Y., and Collins C. (2017). Intrinsic mechanisms for axon regeneration: insights from injured axons in *Drosophila*. Curr Opin Genet Dev 44, 84–91. 28232273 10.1016/j.gde.2017.01.009 PMC5447494

[31] Brace E.J., and DiAntonio A. (2017). Models of axon regeneration in *Drosophila*. Exp Neurol 287, 310–317. 26996133 10.1016/j.expneurol.2016.03.014 PMC5026866

[32] Varadarajan S.G., Hunyara J.L., Hamilton N.R., Kolodkin A.L., and Huberman A.D. (2022). Central nervous system regeneration. Cell 185, 77–94. 34995518 10.1016/j.cell.2021.10.029 PMC10896592

[33] Quraishe S., Forbes L.H., and Andrews M.R. (2018). The extracellular environment of the CNS: Influence on plasticity, sprouting, and axonal regeneration after spinal cord injury. Neural plasticity 2018, 2952386. 29849554 10.1155/2018/2952386 PMC5932463

[34] Hilton B.J., Griffin J.M., Fawcett J.W., and Bradke F. (2024). Neuronal maturation and axon regeneration: unfixing circuitry to enable repair. Nat Rev Neurosci 25, 649–667. 39164450 10.1038/s41583-024-00849-3

[35] Curcio M., and Bradke F. (2018). Axon regeneration in the central nervous system: Facing the challenges from the inside. Annu Rev Cell Dev Biol 34, 495–521. 30044649 10.1146/annurev-cellbio-100617-062508

[36] Sulston J.E., Schierenberg E., White J.G., and Thomson J.N. (1983). The embryonic cell lineage of the nematode *Caenorhabditis elegans*. Dev. Biol. 100, 64–119. 6684600 10.1016/0012-1606(83)90201-4

[37] Nechipurenko I.V., Berciu C., Sengupta P., and Nicastro D. (2017). Centriolar remodeling underlies basal body maturation during ciliogenesis in *Caenorhabditis elegans*. Elife 6, e25686. 28411364 10.7554/eLife.25686 PMC5392363

[38] Serwas D., Su T.Y., Roessler M., Wang S., and Dammermann A. (2017). Centrioles initiate cilia assembly but are dispensable for maturation and maintenance in *C. elegans*. J Cell Biol, 1659–1671. 28411189 10.1083/jcb.201610070 PMC5461022

[39] Fujiwara M., Ishihara T., and Katsura I. (1999). A novel WD40 protein, CHE-2, acts cell-autonomously in the formation of *C. elegans* sensory cilia. Development 126, 4839–4848. 10518500 10.1242/dev.126.21.4839

[40] Jensen V.L., Lambacher N.J., Li C., Mohan S., Williams C.L., Inglis P.N., Yoder B.K., Blacque O.E., and Leroux M.R. (2018). Role for intraflagellar transport in building a functional transition zone. EMBO Rep 19, e45862. 30429209 10.15252/embr.201845862 PMC6280794

[41] Xu K., and Ou G. (2022). Cilia regeneration requires an RNA splicing factor from the ciliary base. Cell Regen 11, 29. 36180752 10.1186/s13619-022-00130-x PMC9525525

[42] Swoboda P., Adler H.T., and Thomas J.H. (2000). The RFX-type transcription factor DAF-19 regulates sensory neuron cilium formation in *C. elegans*. Mol Cell 5, 411–421. 10882127 10.1016/s1097-2765(00)80436-0

[43] Kozminski K.G., Johnson K.A., Forscher P., and Rosenbaum J.L. (1993). A motility in the eukaryotic flagellum unrelated to flagellar beating. Proc Natl Acad Sci USA 90, 5519–5523. 8516294 10.1073/pnas.90.12.5519 PMC46752

[44] Rosenbaum J.L., and Witman G.B. (2002). Intraflagellar transport. Nat Rev Mol Cell Biol 3, 813–825. 12415299 10.1038/nrm952

[45] Hammarlund M., Nix P., Hauth L., Jorgensen E.M., and Bastiani M. (2009). Axon regeneration requires a conserved MAP kinase pathway. Science 323, 802–806. 19164707 10.1126/science.1165527 PMC2729122

[46] Yan D., Wu Z., Chisholm A.D., and Jin Y. (2009). The DLK-1 kinase promotes mRNA stability and local translation in *C. elegans* synapses and axon regeneration. Cell 138, 1005–1018. 19737525 10.1016/j.cell.2009.06.023 PMC2772821

[47] Yanik M.F., Cinar H., Cinar H.N., Chisholm A.D., Jin Y., and Ben-Yakar A. (2004). Neurosurgery: functional regeneration after laser axotomy. Nature 432, 822. 15602545 10.1038/432822a

[48] Wu Z., Ghosh-Roy A., Yanik M.F., Zhang J.Z., Jin Y., and Chisholm A.D. (2007). *Caenorhabditis elegans* neuronal regeneration is influenced by life stage, ephrin signaling, and synaptic branching. Proc Natl Acad Sci USA 104, 15132–15137. 17848506 10.1073/pnas.0707001104 PMC1975853

[49] Ward S., Thomson N., White J.G., and Brenner S. (1975). Electron microscopical reconstruction of the anterior sensory anatomy of the nematode *Caenorhabditis elegans*. J Comp Neurol 160, 313–337. 1112927 10.1002/cne.901600305

[50] Doroquez D.B., Berciu C., Anderson J.R., Sengupta P., and Nicastro D. (2014). A high-resolution morphological and ultrastructural map of anterior sensory cilia and glia in C. elegans. eLife 3, e01948. 24668170 10.7554/eLife.01948 PMC3965213

[51] Hammarlund M., Jorgensen E.M., and Bastiani M.J. (2007). Axons break in animals lacking beta-spectrin. J Cell Biol 176, 269–275. 17261846 10.1083/jcb.200611117 PMC2063953

[52] Nix P., Hammarlund M., Hauth L., Lachnit M., Jorgensen E.M., and Bastiani M. (2014). Axon regeneration genes identified by RNAi screening in *C. elegans*. J Neurosci 34, 629–645. 24403161 10.1523/JNEUROSCI.3859-13.2014 PMC3870940

[53] Signor D., Wedaman K.P., Rose L.S., and Scholey J.M. (1999). Two heteromeric kinesin complexes in chemosensory neurons and sensory cilia of *Caenorhabditis elegans*. Mol Biol Cell 10, 345–360. 9950681 10.1091/mbc.10.2.345 PMC25173

[54] Snow J.J., Ou G., Gunnarson A.L., Walker M.R., Zhou H.M., Brust-Mascher I., and Scholey J.M. (2004). Two anterograde intraflagellar transport motors cooperate to build sensory cilia on *C. elegans* neurons. Nat Cell Biol 6, 1109–1113. 15489852 10.1038/ncb1186

[55] Philbrook A., O'Donnell M.P., Grunenkovaite L., and Sengupta P. (2024). Cilia structure and intraflagellar transport differentially regulate sensory response dynamics within and between *C. elegans* chemosensory neurons. PLoS Biol 22, e3002892. 39591402 10.1371/journal.pbio.3002892 PMC11593760

[56] Troemel E.R., Chou J.H., Dwyer N.D., Colbert H.A., and Bargmann C.I. (1995). Divergent seven transmembrane receptors are candidate chemosensory receptors in *C. elegans*. Cell 83, 207–218. 7585938 10.1016/0092-8674(95)90162-0

[57] Bargmann C.I., and Horvitz H.R. (1991). Chemosensory neurons with overlapping functions direct chemotaxis to multiple chemicals in *C. elegans*. Neuron 7, 729–742. 1660283 10.1016/0896-6273(91)90276-6

[58] Culotti J.G., and Russell R.L. (1978). Osmotic avoidance defective mutants of the nematode *Caenorhabditis elegans*. Genetics 90, 243–256. 730048 10.1093/genetics/90.2.243 PMC1213887

[59] Efimenko E., Bubb K., Mak H.Y., Holzman T., Leroux M.R., Ruvkun G., Thomas J.H., and Swoboda P. (2005). Analysis of *xbx* genes in *C. elegans*. Development 132, 1923–1934. 15790967 10.1242/dev.01775

[60] Chen N., Mah A., Blacque O.E., Chu J., Phgora K., Bakhoum M.W., Newbury C.R., Khattra J., Chan S., Go A., (2006). Identification of ciliary and ciliopathy genes in *Caenorhabditis elegans* through comparative genomics. Genome Biol 7, R126. 17187676 10.1186/gb-2006-7-12-r126 PMC1794439

[61] Blacque O.E., Perens E.A., Boroevich K.A., Inglis P.N., Li C., Warner A., Khattra J., Holt R.A., Ou G., Mah A.K., (2005). Functional genomics of the cilium, a sensory organelle. Curr Biol 15, 935–941. 15916950 10.1016/j.cub.2005.04.059

[62] De Stasio E.A., Mueller K.P., Bauer R.J., Hurlburt A.J., Bice S.A., Scholtz S.L., Phirke P., Sugiaman-Trapman D., Stinson L.A., Olson H.B., (2018). An expanded role for the RFX transcription factor DAF-19, with dual functions in ciliated and nonciliated neurons. Genetics 208, 1083–1097. 29301909 10.1534/genetics.117.300571 PMC5844324

[63] Zhang L., Ward J.D., Cheng Z., and Dernburg A.F. (2015). The auxin-inducible degradation (AID) system enables versatile conditional protein depletion in *C. elegans*. Development 142, 4374–4384. 26552885 10.1242/dev.129635 PMC4689222

[64] Nishimura K., Fukagawa T., Takisawa H., Kakimoto T., and Kanemaki M. (2009). An auxin-based degron system for the rapid depletion of proteins in nonplant cells. Nat Methods 6, 917–922. 19915560 10.1038/nmeth.1401

[65] Senti G., and Swoboda P. (2008). Distinct isoforms of the RFX transcription factor DAF-19 regulate ciliogenesis and maintenance of synaptic activity. Mol Biol Cell 19, 5517–5528. 18843046 10.1091/mbc.E08-04-0416 PMC2592639

[66] Ahn S., Yang H., Son S., Lee H.S., Park D., Yim H., Choi H.J., Swoboda P., and Lee J. (2022). The C. elegans regulatory factor X (RFX) DAF-19M module: A shift from general ciliogenesis to cell-specific ciliary and behavioral specialization. Cell Rep 39, 110661. 35417689 10.1016/j.celrep.2022.110661

[67] Wang J., Schwartz H.T., and Barr M.M. (2010). Functional specialization of sensory cilia by an RFX transcription factor isoform. Genetics 186, 1295–1307. 20923979 10.1534/genetics.110.122879 PMC2998312

[68] Rosenbaum J.L., Moulder J.E., and Ringo D.L. (1969). Flagellar elongation and shortening in *Chlamydomonas*. The use of cycloheximide and colchicine to study the synthesis and assembly of flagellar proteins. J Cell Biol 41, 600–619. 5783876 10.1083/jcb.41.2.600 PMC2107765

[69] Lefebvre P.A., and Rosenbaum J.L. (1986). Regulation of the synthesis and assembly of ciliary and flagellar proteins during regeneration. Annu Rev Cell Biol 2, 517–546. 3548775 10.1146/annurev.cb.02.110186.002505

[70] Lefebvre P.A., Silflow C.D., Wieben E.D., and Rosenbaum J.L. (1980). Increased levels of mRNAs for tubulin and other flagellar proteins after amputation or shortening of *Chlamydomonas* flagella. Cell 20, 469–477. 6156007 10.1016/0092-8674(80)90633-9

[71] Baker E.J., Schloss J.A., and Rosenbaum J.L. (1984). Rapid changes in tubulin RNA synthesis and stability induced by deflagellation in *Chlamydomonas*. J Cell Biol 99, 2074–2081. 6209288 10.1083/jcb.99.6.2074 PMC2113547

[72] Schloss J.A., Silflow C.D., and Rosenbaum J.L. (1984). mRNA abundance changes during flagellar regeneration in *Chlamydomonas reinhardtii*. Mol Cell Biol 4, 424–434. 6546968 10.1128/mcb.4.3.424-434.1984 PMC368719

[73] Perlaza K., Zamora I., and Marshall W.F. (2022). Role of intraflagellar transport in transcriptional control during flagellar regeneration in *Chlamydomonas*. Mol Biol Cell, mbcE22090444. 10.1091/mbc.E22-09-0444 PMC10208099 36542488

[74] Stolc V., Samanta M.P., Tongprasit W., and Marshall W.F. (2005). Genome-wide transcriptional analysis of flagellar regeneration in *Chlamydomonas reinhardtii* identifies orthologs of ciliary disease genes. Proc Natl Acad Sci USA 102, 3703–3707. 15738400 10.1073/pnas.0408358102 PMC553310

[75] Lacey S.E., Foster H.E., and Pigino G. (2023). The molecular structure of IFT-A and IFT-B in anterograde intraflagellar transport trains. Nat Struct Mol Biol 30, 584–593. 36593313 10.1038/s41594-022-00905-5 PMC10191852

[76] Taschner M., Kotsis F., Braeuer P., Kuehn E.W., and Lorentzen E. (2014). Crystal structures of IFT70/52 and IFT52/46 provide insight into intraflagellar transport B core complex assembly. J Cell Biol 207, 269–282. 25349261 10.1083/jcb.201408002 PMC4210449

[77] Perkins L.A., Hedgecock E.M., Thomson J.N., and Culotti J.G. (1986). Mutant sensory cilia in the nematode *Caenorhabditis elegans*. Dev Biol 117, 456–487. 2428682 10.1016/0012-1606(86)90314-3

[78] Ou G., Blacque O.E., Snow J.J., Leroux M.R., and Scholey J.M. (2005). Functional coordination of intraflagellar transport motors. Nature 436, 583–587. 16049494 10.1038/nature03818

[79] Tedeschi A., and Bradke F. (2013). The DLK signalling pathway--a double-edged sword in neural development and regeneration. EMBO Rep 14, 605–614. 23681442 10.1038/embor.2013.64 PMC3701236

[80] Jin Y., and Zheng B. (2019). Multitasking: dual leucine zipper-bearing kinases in neuronal development and stress management. Annu Rev Cell Dev Biol 35, 501–521. 31590586 10.1146/annurev-cellbio-100617-062644 PMC7015696

[81] Valakh V., Frey E., Babetto E., Walker L.J., and DiAntonio A. (2015). Cytoskeletal disruption activates the DLK/JNK pathway, which promotes axonal regeneration and mimics a preconditioning injury. Neurobiol Dis 77, 13–25. 25726747 10.1016/j.nbd.2015.02.014 PMC4402261

[82] Hirai S., Banba Y., Satake T., and Ohno S. (2011). Axon formation in neocortical neurons depends on stage-specific regulation of microtubule stability by the dual leucine zipper kinase-c-Jun N-terminal kinase pathway. J Neurosci 31, 6468–6480. 21525288 10.1523/JNEUROSCI.5038-10.2011 PMC6622676

[83] Bounoutas A., Kratz J., Emtage L., Ma C., Nguyen K.C., and Chalfie M. (2011). Microtubule depolymerization in *Caenorhabditis elegans* touch receptor neurons reduces gene expression through a p38 MAPK pathway. Proc Natl Acad Sci USA 108, 3982–3987. 21368137 10.1073/pnas.1101360108 PMC3054000

[84] Ghosh-Roy A., Goncharov A., Jin Y., and Chisholm A.D. (2012). Kinesin-13 and tubulin posttranslational modifications regulate microtubule growth in axon regeneration. Dev Cell 23, 716–728. 23000142 10.1016/j.devcel.2012.08.010 PMC3477258

[85] Sun Y., and Jin Y. (2023). An intraflagellar transport dependent negative feedback regulates the MAPKKK DLK-1 to protect cilia from degeneration. Proc Natl Acad Sci USA 120, e2302801120. 37722038 10.1073/pnas.2302801120 PMC10523469

[86] van der Vaart A., Rademakers S., and Jansen G. (2015). DLK-1/p38 MAP Kinase signaling controls cilium length by regulating RAB-5 mediated endocytosis in *Caenorhabditis elegans*. PLoS Genet 11, e1005733. 26657059 10.1371/journal.pgen.1005733 PMC4686109

[87] Bates S.G., Harris N., and Sengupta P. (2026). A repressive regulatory cascade shapes temporal patterning of activity-regulated gene expression in a defined sensory neuron type. bioRxiv. 10.64898/2026.05.14.725236.

[88] Bacaj T., Tevlin M., Lu Y., and Shaham S. (2008). Glia are essential for sensory organ function in *C. elegans*. Science 322, 744–747. 18974354 10.1126/science.1163074 PMC2735448

[89] Varandas K.C., Hodges B.M., Lubeck L., Farinas A., Liang Y., Lu Y., and Shaham S. (2025). Glia detect and transiently protect against dendrite substructure disruption in *C. elegans*. Nat Commun 16, 79. 39747235 10.1038/s41467-024-55674-0 PMC11696001

[90] Singhvi A., Liu B., Friedman C.J., Fong J., Lu Y., Huang X.Y., and Shaham S. (2016). A glial K/Cl transporter controls neuronal receptive ending shape by chloride inhibition of an rGC. Cell 165, 936–948. 27062922 10.1016/j.cell.2016.03.026 PMC4860081

[91] Wallace S.W., Singhvi A., Liang Y., Lu Y., and Shaham S. (2016). PROS-1/Prospero is a major regulator of the glia-specific secretome controlling sensory-neuron shape and function in *C. elegans*. Cell Rep 15, 550–562. 27068465 10.1016/j.celrep.2016.03.051 PMC4838487

[92] Chamberlain K.L., Miller S.H., and Keller L.R. (2008). Gene expression profiling of flagellar disassembly in *Chlamydomonas reinhardtii*. Genetics 179, 7–19. 18493036 10.1534/genetics.107.082149 PMC2390646

[93] Remillard S.P., and Witman G.B. (1982). Synthesis, transport, and utilization of specific flagellar proteins during flagellar regeneration in *Chlamydomonas*. J Cell Biol 93, 615–631. 7118994 10.1083/jcb.93.3.615 PMC2112125

[94] Tyssowski K.M., DeStefino N.R., Cho J.H., Dunn C.J., Poston R.G., Carty C.E., Jones R.D., Chang S.M., Romeo P., Wurzelmann M.K., (2018). Different neuronal activity patterns induce different gene expression programs. Neuron 98, 530–546 e511. 29681534 10.1016/j.neuron.2018.04.001 PMC5934296

[95] Yap E.L., and Greenberg M.E. (2018). Activity-regulated transcription: Bridging the gap between neural activity and behavior. Neuron 100, 330–348. 30359600 10.1016/j.neuron.2018.10.013 PMC6223657

[96] Pumo G., and Rijli F.M. (2025). Chromatin regulation of neuronal activity-dependent gene programs in circuit formation and plasticity. Curr Opin Neurobiol 92, 103024. 40262385 10.1016/j.conb.2025.103024

[97] Cheshire J.L., and Keller L.R. (1991). Uncoupling of *Chlamydomonas* flagellar gene expression and outgrowth from flagellar excision by manipulation of Ca2+. J Cell Biol 115, 1651–1659. 1757467 10.1083/jcb.115.6.1651 PMC2289195

[98] Quader H., Cherniack J., and Filner P. (1978). Participation of calcium in flagellar shortening and regeneration in *Chlamydomonas reinhardii*. Exp Cell Res 113, 295–301. 233767 10.1016/0014-4827(78)90369-5

[99] Xie Y., Moussaif M., Choi S., Xu L., and Sze J.Y. (2013). RFX transcription factor DAF-19 regulates 5-HT and innate immune responses to pathogenic bacteria in *Caenorhabditis elegans*. PLoS Genet 9, e1003324. 23505381 10.1371/journal.pgen.1003324 PMC3591283

[100] Lai J., Demirbas D., Phillips K., Zhao B., Wallace H., Seferian M., Nakayama T., Harris H., Chatzipli A., Lee E.A., and Yu T.W. (2025). Multi-omic analysis of the ciliogenic transcription factor RFX3 reveals a role in promoting activity-dependent responses via enhancing CREB binding in human neurons. bioRxiv. 10.1101/2025.02.27.640588.

[101] Neumann S., Achiro J.M., Watanabe M., Bonnano S.L., Deng W., Wohlschlegel J.A., and Martin K.C. (2025). Cytoplasmic and nuclear protein interaction networks of the synapto-nuclear messenger CRTC1 in neurons reveal cooperative chromatin binding between CREB1 and CRTC1, MEF2C and RFX3. bioRxiv. 10.1101/2025.07.02.662820.

[102] Chen B., Niu J., Kreuzer J., Zheng B., Jarugumilli G.K., Haas W., and Wu X. (2018). Auto-fatty acylation of transcription factor RFX3 regulates ciliogenesis. Proc Natl Acad Sci USA 115, E8403–E8412. 30127002 10.1073/pnas.1800949115 PMC6130365

[103] Moreno C.S., Rogers E.M., Brown J.A., and Boss J.M. (1997). Regulatory factor X, a bare lymphocyte syndrome transcription factor, is a multimeric phosphoprotein complex. J Immunol 158, 5841–5848. 9190936

[104] Brocal-Ruiz R., Esteve-Serrano A., Mora-Martinez C., Franco-Rivadeneira M.L., Swoboda P., Tena J.J., Vilar M., and Flames N. (2023). Forkhead transcription factor FKH-8 cooperates with RFX in the direct regulation of sensory cilia in *Caenorhabditis elegans*. Elife 12, e89702. 37449480 10.7554/eLife.89702 PMC10393296

[105] Brown J.M. (2025). Cilia and transcription: a mini review. Frontiers Cell Dev Biol 13, 1582796. 10.3389/fcell.2025.1582796 PMC12183034 40552307

[106] Choksi S.P., Lauter G., Swoboda P., and Roy S. (2014). Switching on cilia: transcriptional networks regulating ciliogenesis. Development 141, 1427–1441. 24644260 10.1242/dev.074666

[107] Starich T.A., Herman R.K., Kari C.K., Yeh W.-H., Schackwitz W.S., Schuyler M.W., Collet J., Thomas J.H., and Riddle D.L. (1995). Mutations affecting the chemosensory neurons of *Caenorhabditis elegans*. Genetics 139, 171–188. 7705621 10.1093/genetics/139.1.171 PMC1206316

[108] Ou G., Koga M., Blacque O.E., Murayama T., Ohshima Y., Schafer J.C., Li C., Yoder B.K., Leroux M.R., and Scholey J.M. (2007). Sensory ciliogenesis in *Caenorhabditis elegans*: assignment of IFT components into distinct modules based on transport and phenotypic profiles. Mol Biol Cell 18, 1554–1569. 17314406 10.1091/mbc.E06-09-0805 PMC1855012

[109] Zhou J., Feng C., Sun Y., Noma K., and Jin Y. (2025). A tubulin-MAPKKK pathway engages tubulin isotype interaction for neuroprotection. Proc Natl Acad Sci USA 122, e2507208122. 40811477 10.1073/pnas.2507208122 PMC12403078

[110] Sun Y., Zhou J., Debnath A., Xie B., Wang Z., and Jin Y. (2025). Multiple regulators constrain the abundance of Caenorhabditis elegans DLK-1 in ciliated sensory neurons. G3 15. 10.1093/g3journal/jkaf004 PMC11917482 39854273

[111] Zhang X., Liu J., Pan T., Ward A., Liu J., and Xu X.Z.S. (2022). A cilia-independent function of BBSome mediated by DLK-MAPK signaling in *C. elegans* photosensation. Dev Cell 57, 1545–1557 e1544. 35649417 10.1016/j.devcel.2022.05.005 PMC9233093

[112] Singhvi A., Shaham S., and Rapti G. (2024). Glia development and function in the nematode *Caenorhabditis elegans*. Cold Spring Harb Perspect Biol 16, a041346. 38565269 10.1101/cshperspect.a041346 PMC11445397

[113] Razzauti A., and Laurent P. (2021). Ectosome uptake by glia sculpts *Caenorhabditis elegans* sensory cilia. eLife 10, e67670.34533135

[114] Ray S., Gurung P., Manning R.S., Kravchuk A.A., and Singhvi A. (2024). Neuron cilia restrain glial KCC-3 to a microdomain to regulate multisensory processing. Cell Rep 43, 113844. 38421867 10.1016/j.celrep.2024.113844 PMC11296322

[115] Perens E.A., and Shaham S. (2005). *C. elegans daf-6* encodes a patched-related protein required for lumen formation. Dev Cell 8, 893–906. 15935778 10.1016/j.devcel.2005.03.009

[116] Yoshida A., Nakano S., Suzuki T., Ihara K., Higashiyama T., and Mori I. (2015). A glial K /Cl cotransporter modifies temperature-evoked dynamics in *C. elegans* sensory neurons. Genes Brain Behav 15, 429–440. 26463820 10.1111/gbb.12260

[117] Raiders S., Black E.C., Bae A., MacFarlane S., Klein M., Shaham S., and Singhvi A. (2021). Glia actively sculpt sensory neurons by controlled phagocytosis to tune animal behavior. Elife 10, e63532. 33759761 10.7554/eLife.63532 PMC8079151

[118] Wu J., Yarmey V.R., Yang O.J., Soderblom E.J., San-Miguel A., and Yan D. (2025). Heat shock proteins function as signaling molecules to mediate neuron-glia communication in *C. elegans* during aging. Nat Neurosci 28, 1635–1648. 40533573 10.1038/s41593-025-01989-0

[119] Ferguson T.A., and Son Y.J. (2011). Extrinsic and intrinsic determinants of nerve regeneration. J Tissue Eng 2, 2041731411418392. 22292105 10.1177/2041731411418392 PMC3251917

[120] Yeon J., Kim J., Sato K., Nurrish S., Chen L., Krishnan N., Bates S., Ihara S., Rasouli S., Porwal C., (2026). An enteric neuron ionotropic receptor regulates salt stress resistance. Nature, doi: 10.1038/s41586-026-10348-3. PMC13293861 41922765

[121] Harris N., Dutta P., Krishnan N., Nurrish S., Wilder E.C., and Sengupta P. (2026). Experience-dependent reconfiguration of thermoreceptors regulates neuronal response plasticity. Curr Biol 4, 1076–1085. 10.1016/j.cub.2025.12.054 PMC12866963 41610847

[122] Dokshin G.A., Ghanta K.S., Piscopo K.M., and Mello C.C. (2018). Robust genome editing with short single-stranded and long, partially single-stranded DNA donors in *Caenorhabditis elegans*. Genetics 210, 781–787. 30213854 10.1534/genetics.118.301532 PMC6218216

[123] Chronis N., Zimmer M., and Bargmann C.I. (2007). Microfluidics for *in vivo* imaging of neuronal and behavioral activity in *Caenorhabditis elegans*. Nat Methods 4, 727–731. 17704783 10.1038/nmeth1075

[124] Khan M., Hartmann A.H., O’Donnell M.P., Piccione M., Pandey A., Chao P.-H., Dwyer N.D., Bargmann C.I., and Sengupta P. (2022). Context-dependent reversal of odorant preference is driven by inversion of the response in a single sensory neuron type. PLoS Biol 20, e3001677. 35696430 10.1371/journal.pbio.3001677 PMC9232122

[125] Byrne A.B., and Hammarlund M. (2017). Axon regeneration in *C. elegans*: Worming our way to mechanisms of axon regeneration. Exp Neurol 287, 300–309. 27569538 10.1016/j.expneurol.2016.08.015 PMC5136328

