# Supplementary material for: Neuron-intrinsic and glial pathways regulate sensory cilia regeneration in adult C. elegans
Source: bioRxiv. 2026 Jul 10:2026.05.15.725580. Originally published 2026 May 17. Preprint. [Version 2] doi: 10.64898/2026.05.15.725580 (PMC13192683; doi:10.64898/2026.05.15.725580)
Supplement: 1 [file NIHPP2026.05.15.725580V2-supplement-1.pdf]

bioRxiv preprint doi: https://doi.org/10.64898/2026.05.15.725580; this version posted July 10, 2026. The copyright holder for this preprint (which was not certified by peer review) is the author/funder, who has granted bioRxiv a license to display the preprint in perpetuity. It is made available under a CC-BY 4.0 International license.

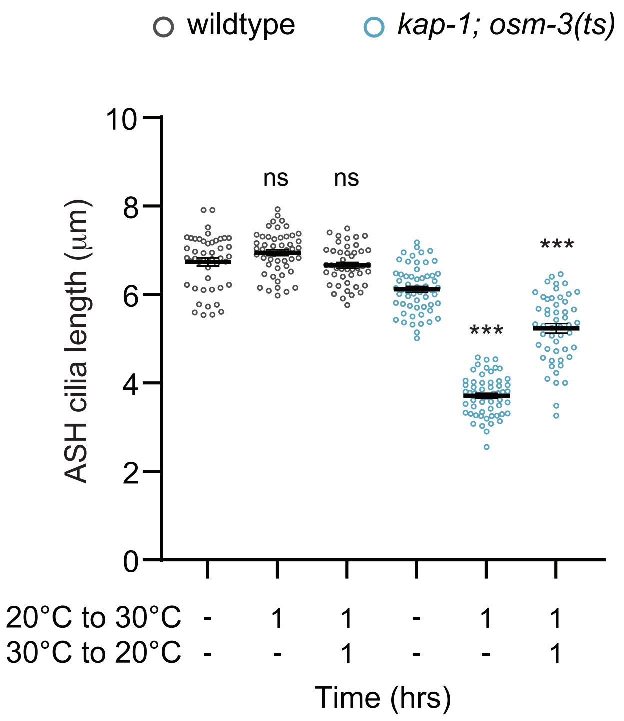

Figure S1, related to Figure 1. ASH cilia truncate and regrow rapidly in the adult.
Quantification of ASH cilia length in wildtype (black) and *kap-1(ok676); osm-3(oy156ts)* (blue) mutants at the indicated temperature shift conditions. Each circle is the length of a single ASH cilium. ***: different at $P<0.001$ from unshifted within each genotype; ns: not significant (one-way ANOVA with Tukey's multiple comparison test). Errors are SEM. n≥44 each; 3 independent experiments.

bioRxiv preprint doi: https://doi.org/10.64898/2026.05.15.725580; this version posted July 10, 2026. The copyright holder for this preprint (which was not certified by peer review) is the author/funder, who has granted bioRxiv a license to display the preprint in perpetuity. It is made available under a CC-BY 4.0 International license.

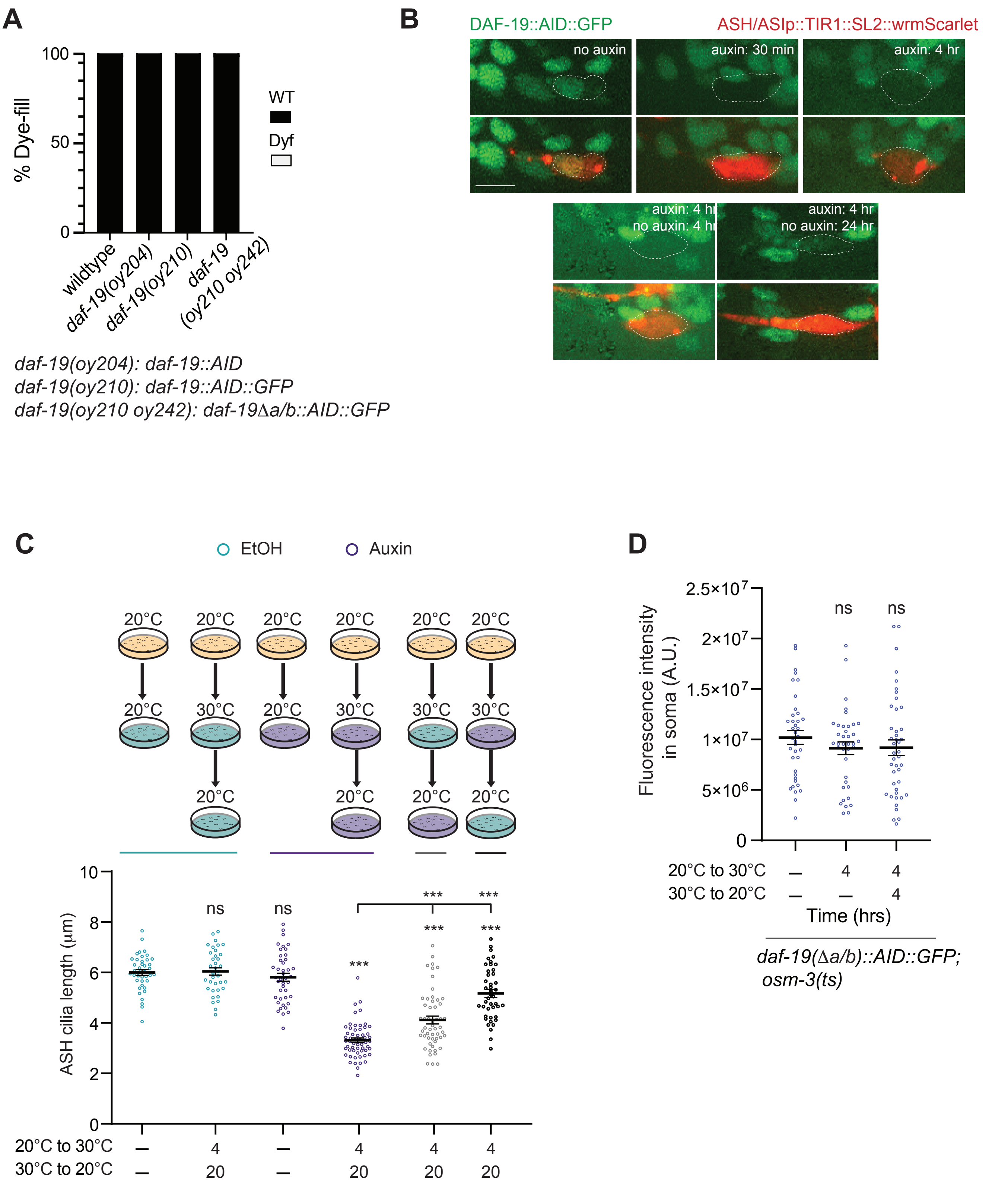

Figure S2, related to Figures 2 and 3. DAF-19 is required for cilia regeneration but is dispensable for maintenance.

A) Percentage of animals of the indicated genotypes in which a subset of ciliated neurons including ASH/ASI are filled with dye. n>300 animals each; 3 independent experiments. Dyf: dye-filling defective.

B) Representative images of endogenously tagged DAF-19::AID::GFP expression in ASH/ASI neurons in the absence or presence of auxin as indicated. These neurons also express *sra-6*p*::TIR1::SL2::wrmScarlet* from an extrachromosomal array. ASH/ASI soma are indicated with dashed outlines. Scale bar: 5 µm.

bioRxiv preprint doi: https://doi.org/10.64898/2026.05.15.725580; this version posted July 10, 2026. The copyright holder for this preprint (which was not certified by peer review) is the author/funder, who has granted bioRxiv a license to display the preprint in perpetuity. It is made available under a CC-BY 4.0 International license.

C) (Top) Schematic of experimental conditions for ASH cilia length quantifications (bottom). Each circle is the length of a single ASH cilium. ***: different at $P<0.001$ from unshifted or between indicated; ns: not significant (one-way ANOVA with Tukey's multiple comparison test). Errors are SEM. n≥36 each; 3 independent experiments.

D) Quantification of reporter fluorescence intensity in ASH/ASI soma of endogenously reporter-tagged *daf-19(oy210 oy242Δa/b)* in *osm-3(oy156ts)* animals in the shown temperature shift conditions. Each circle is the value from an ASH and/or ASI soma in one amphid organ. ns: not significant from unshifted (one-way ANOVA with Tukey multiple comparison's test). Errors are SEM. n≥38 each; 2 independent experiments.

bioRxiv preprint doi: https://doi.org/10.64898/2026.05.15.725580; this version posted July 10, 2026. The copyright holder for this preprint (which was not certified by peer review) is the author/funder, who has granted bioRxiv a license to display the preprint in perpetuity. It is made available under a CC-BY 4.0 International license.

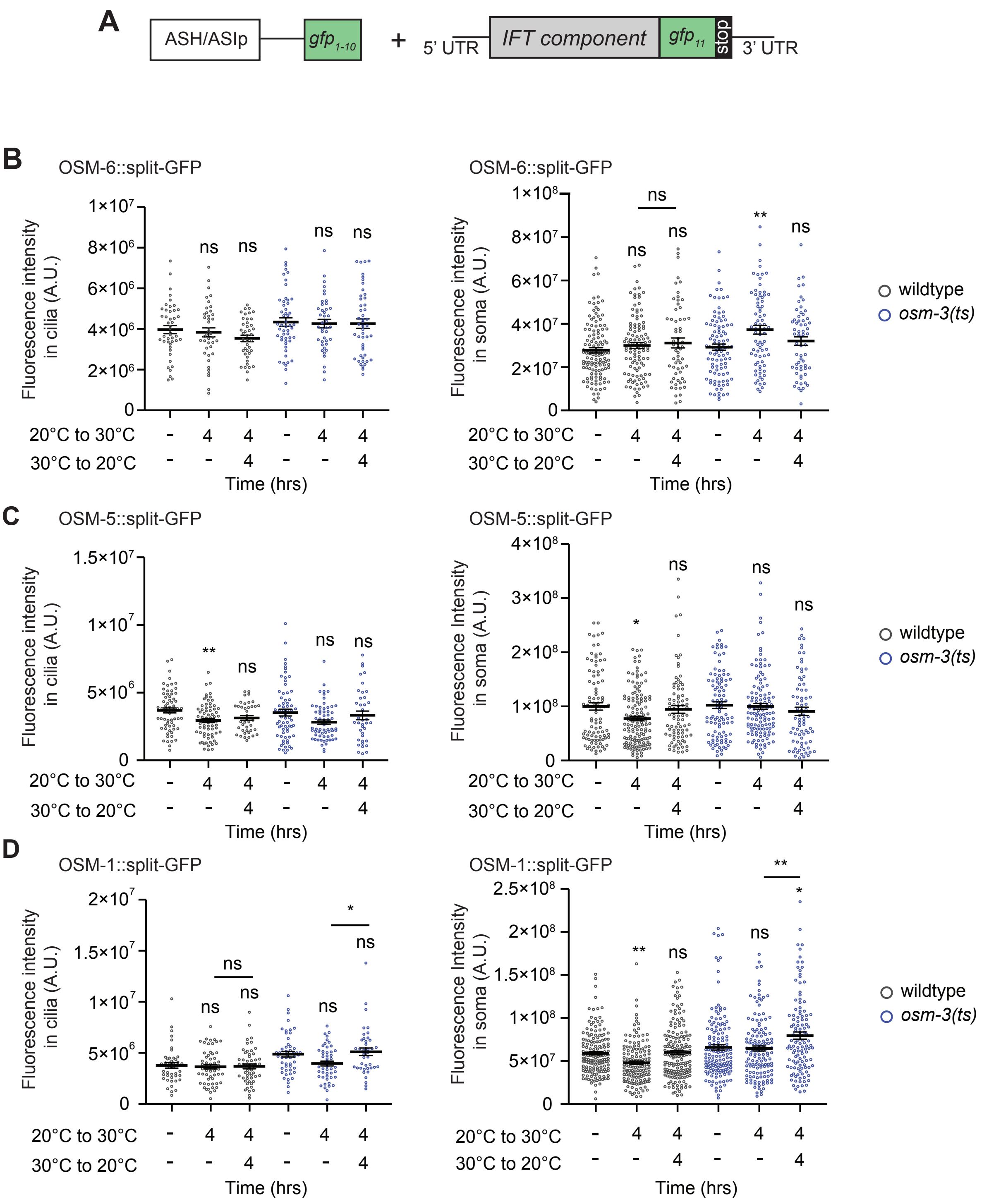

Figure S3, related to Figure 4. IFT protein levels in ASH soma are altered upon cilia truncation and regeneration.

A) Schematic of endogenously tagged translational reporter expression.

B-D) Quantification of fluorescence intensities in ASH/ASI cilia (left) and soma (right) of wildtype (black) or *osm-3(oy156ts)* (blue) animals expressing the indicated IFT translational reporters in the shown temperature shift conditions. Each circle is the value from an ASH and/or ASI cilia (left) or soma (right) in an amphid organ. * and **: different at $P<0.05$ and 0.01, respectively, from unshifted within each genotype or between indicated; ns: not significant (one-way ANOVA with Tukey's multiple comparison test or unpaired Welch's t-test). Errors are SEM. n≥39 each; >3 independent experiments.

bioRxiv preprint doi: https://doi.org/10.64898/2026.05.15.725580; this version posted July 10, 2026. The copyright holder for this preprint (which was not certified by peer review) is the author/funder, who has granted bioRxiv a license to display the preprint in perpetuity. It is made available under a CC-BY 4.0 International license.

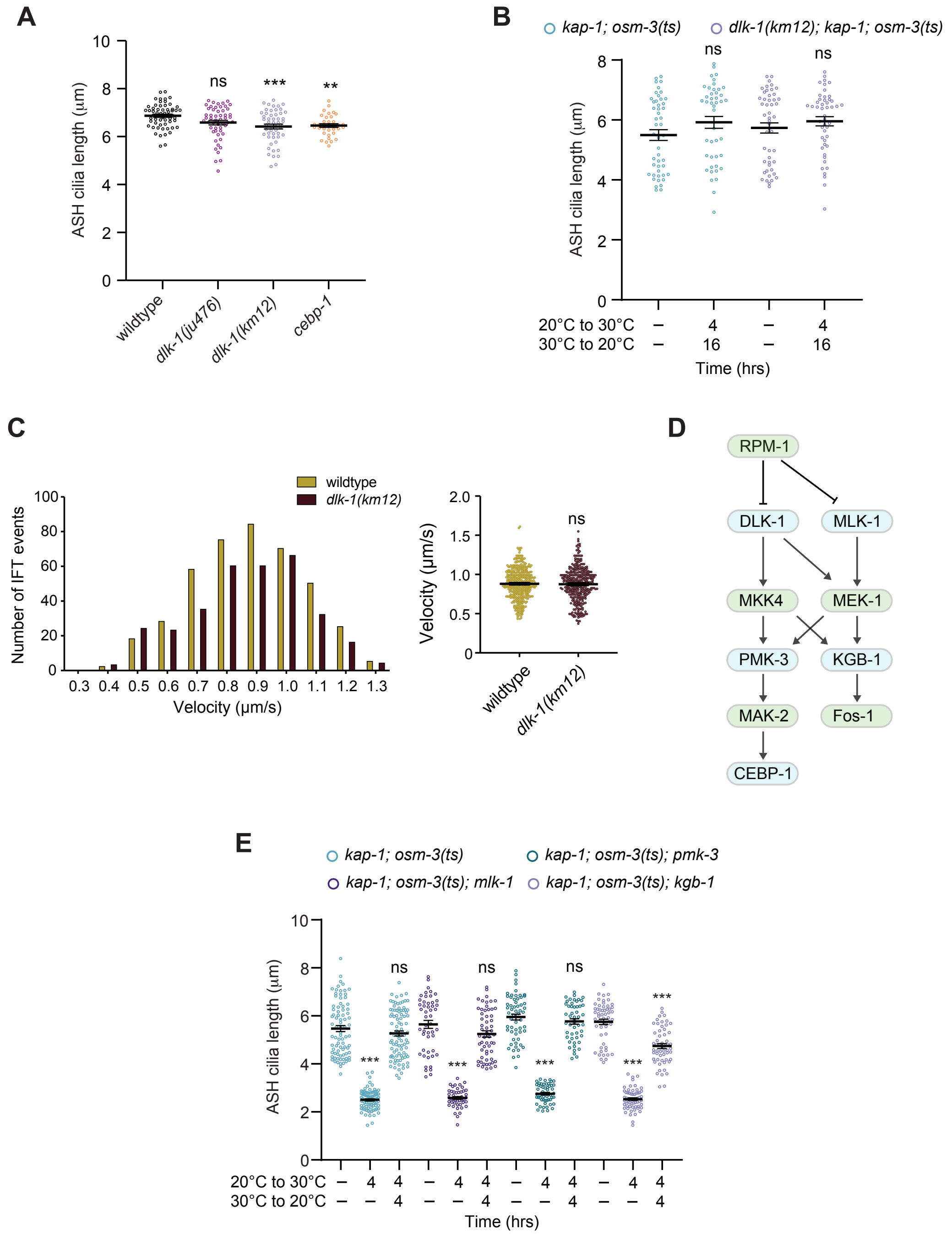

Figure S4, related to Figure 5. DLK-1 and CEBP-1 are necessary for efficient cilia regeneration.

A) Quantification of ASH cilia length in the shown genetic backgrounds Each circle is the length of a single ASH cilium. ** and ***: different at *P*<0.01 and 0.001 from wildtype; ns: not significant (one-way ANOVA with Tukey's multiple comparison test). Errors are SEM. n≥35 each; 2 independent experiments.

B,E) Quantification of ASH cilia length in the shown genetic backgrounds at the indicated temperature shift conditions. Each circle is the length of a single ASH cilium. ***: different at *P*<0.001 from unshifted within each genotype; ns: not significant (B: unpaired Welch's t-test, E: one-way ANOVA with Tukey's multiple comparison test). Errors are SEM. n≥44 each; 3 independent experiments.

C) Histogram (left) and quantification (right) of velocities of reconstituted endogenously tagged OSM-6::split-GFP in ASH cilia in wildtype and *dlk-1(km12)* animals. n≥24 animals per genotype; 3 independent experiments. ns: not significant (unpaired Welch's t-test).

D) Known DLK-1-regulated signaling pathways. Adapted from [1,2].

bioRxiv preprint doi: https://doi.org/10.64898/2026.05.15.725580; this version posted July 10, 2026. The copyright holder for this preprint (which was not certified by peer review) is the author/funder, who has granted bioRxiv a license to display the preprint in perpetuity. It is made available under a CC-BY 4.0 International license.

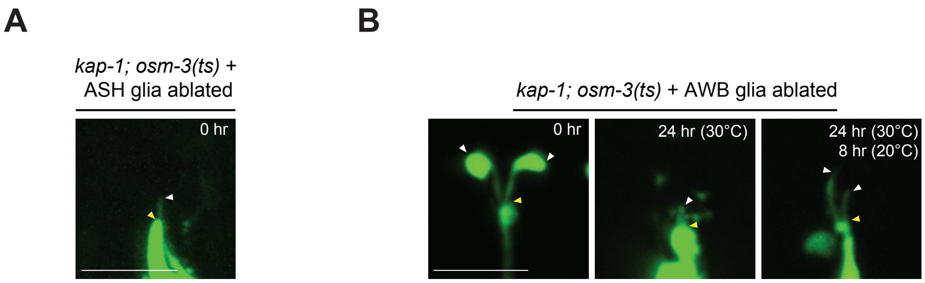

Figure S5, related to Figure 6. Cilia morphology is altered in the absence of glia.
Representative images of ASH (A) and AWB (B) cilia in the indicated genetic backgrounds and temperature shift conditions. Yellow/white arrowheads: cilia base/cilia tip. Scale bars: 5 µm.

bioRxiv preprint doi: https://doi.org/10.64898/2026.05.15.725580; this version posted July 10, 2026. The copyright holder for this preprint (which was not certified by peer review) is the author/funder, who has granted bioRxiv a license to display the preprint in perpetuity. It is made available under a CC-BY 4.0 International license.

Table S1, related to all Figures. List of strains used in this work.

Strain	Genotype	Source
PY1054	oyIs14[sra-6p::gfp]	[3]
PY12030	kap-1(ok676); osm-3(oy156ts); oyIs14[sra-6p::gfp]	[4]
PY12609	osm-3(oy156ts); oyIs14[sra-6p::gfp]	This work
PY12014	kap-1(ok676); osm-3(oy156ts)	[4]
PY12019	kap-1(ok676); osm-3(oy156ts); kyIs602[sra-6p::GCaMP3]	[4]
PY12005	kyIs602[sra-6p::GCaMP3]	CX15832, [5]
PY12006	osm-6(oy166[osm-6::gfp$_{11}$])	[4]
PY12007	osm-6(oy166[osm-6::gfp$_{11}$]); oyEx682[sra-6p::gfp$_{1-10}$; unc-122p::gfp]	[4]
PY12036	kap-1(ok676); osm-3(oy156ts); osm-6(oy166[osm-6::gfp$_{11}$]); oyEx682[sra-6p::gfp$_{1-10}$; unc-122p::gfp]	[4]
PY12610	daf-19(oy204[daf-19::AID]); oyIs14[sra-6p::gfp]	This work
PY12611	daf-19(oy204[daf-19::AID]); oyIs14[sra-6p::gfp]; oyEx830[sra-6p::TIR1::SL2::wrmScarlet]	This work
PY12604	daf-19(oy204[daf-19::AID])	This work
PY12605	daf-19(oy210[daf-19::AID::gfp])	This work
PY13218	daf-19(oy210[daf-19::AID::gfp] oy242[daf-19Δa/b])	This work
PY12612	daf-19(oy204[daf-19::AID]); kap-1(ok676); osm-3(oy156ts); oyIs14[sra-6p::gfp]; oyEx830[sra-6p::TIR1::SL2::wrmScarlet]	This work
PY12613	daf-19(oy210[daf-19::AID::gfp]); oyEx830[sra-6p::TIR1::SL2::wrmScarlet]	This work
PY12664	daf-19(oy210[daf-19::AID::gfp] oy225[daf-19c::mScarlet-I3]); oyEx831[sra-6p::eBfp; unc-122p::gfp]	This work
PY12665	daf-19(oy210[daf-19::AID::gfp] oy225[daf-19c::mScarlet-I3]); osm-3(oy156ts); oyEx831[sra-6p::eBfp; unc-122p::gfp]	This work
PY12614	daf-19(oy210[daf-19::AID::gfp] oy242[daf-19Δa/b]); oyEx831[sra-6p::eBfp; unc-122p::gfp]	This work
PY12615	daf-19(oy210[daf-19::AID::gfp] oy242[daf-19Δa/b]); kap-1(ok676); osm-3(oy156ts); oyEx831[sra-6p::eBfp; unc-122p::gfp]	This work
PY12616	daf-19(tm5562); kap-1(ok676); osm-3(oy156ts); oyIs14[sra-6p::gfp]	This work
PY12617	daf-19(oy204[daf-19::AID]); kap-1(ok676); osm-3(oy156ts); oyIs14[sra-6p::gfp]; oyEx830[sra-6p::TIR1::SL2::wrmScarlet]; oyEx833[daf-19d genomic; vha-6p::NLS::gfp]	This work
PY12618	daf-19(oy204[daf-19::AID]); kap-1(ok676); osm-3(oy156ts); oyIs14[sra-6p::gfp]; oyEx830[sra-6p::TIR1::SL2::wrmScarlet]; oyEx834[daf-19d genomic; vha-6p::NLS::gfp]	This work
PY12619	osm-3(oy156ts); osm-6(oy166[osm-6::gfp$_{11}$]); oyEx682[sra-6p::gfp$_{1-10}$; unc-122p::gfp]	This work
PY12607	osm-6(oy205[osm-6::SL2::gfp$_{11}$])	This work
PY12620	osm-6(oy205[osm-6::SL2::gfp$_{11}$]); oyEx682[sra-6p::gfp$_{1-10}$; unc-122p::gfp]	This work
PY12621	osm-3(oy156ts); osm-6(oy205[osm-6::SL2:: gfp$_{11}$]); oyEx682[sra-6p::gfp$_{1-10}$; unc-122p::gfp]	This work
PY12622	osm-6(oy166[osm-6::gfp$_{11}$]); osm-5(p813); oyEx682[sra-6p:gfp$_{1-10}$; unc-122p::gfp]	This work
PY12623	osm-3(p802); osm-6(oy166[osm-6::gfp$_{11}$]); oyEx682[sra-6p::gfp$_{1-10}$; unc-122p::gfp]	This work

bioRxiv preprint doi: https://doi.org/10.64898/2026.05.15.725580; this version posted July 10, 2026. The copyright holder for this preprint (which was not certified by peer review) is the author/funder, who has granted bioRxiv a license to display the preprint in perpetuity. It is made available under a CC-BY 4.0 International license.

PY12624	*osm-6*(*oy205*[*osm-6*::*SL2*::*gfp*$_{11}$]); *osm-5*(*p813*); *oyEx682*[*sra-6*p::*gfp*$_{1-10}$; *unc-122*p::*gfp*]	This work
PY12625	*osm-3*(*p802*); *osm-6*(*oy205*[*osm-6*::*SL2*::*gfp*$_{11}$]); *oyEx682*[*sra-6*p::*gfp*$_{1-10}$; *unc-122*p::*gfp*]	This work
PY12626	*osm-1*(*oy226*[*osm-1*::*SL2*::*gfp*$_{11}$])	This work
PY12627	*osm-1*(*oy226*[*osm-1*::*SL2*::*gfp*$_{11}$]); *oyEx682*[*sra-6*p::*gfp*$_{1-10}$; *unc-122*p::*gfp*]	This work
PY12628	*osm-3*(*oy156ts*); *osm-1*(*oy226*[*osm-1*::*SL2*::*gfp*$_{11}$]); *oyEx682*[*sra-6*p::*gfp*$_{1-10}$; *unc-122*p::*gfp*]	This work
PY12600	*osm-1*(*oy176*[*osm-1*::*gfp*$_{11}$])	This work
PY12629	*osm-1*(*oy176*[*osm-1*::*gfp*$_{11}$]); *oyEx682*[*sra-6*p::*gfp*$_{1-10}$; *unc-122*p::*gfp*]	This work
PY12630	*osm-3*(*oy156ts*); *osm-1*(*oy176*[*osm-1*::*gfp*$_{11}$]); *oyEx682*[*sra-6*p::*gfp*$_{1-10}$; *unc-122*p::*gfp*]	This work
PY12631	*osm-6*(*p811*); *osm-1*(*oy176*[*osm-6*::*gfp*$_{11}$]); *oyEx682*[*sra-6*p::*gfp*$_{1-10}$; *unc-122*p::*gfp*]	This work
PY12632	*osm-3*(*p802*); *osm-1*(*oy176*[*osm-1*::*gfp*$_{11}$]); *oyEx682*[*sra-6*p::*gfp*$_{1-10}$; *unc-122*p::*gfp*]	This work
PY12633	*osm-5*(*oy218*[*osm-5*::*SL2*::*gfp*$_{11}$])	This work
PY12634	*osm-5*(*oy218*[*osm-5*::*SL2*::*gfp*$_{11}$]); *oyEx682*[*sra-6*p::*gfp*$_{1-10}$; *unc-122*p::*gfp*]	This work
PY12635	*osm-3*(*oy156ts*); *osm-5*(*oy218*[*osm-5*::*SL2*::*gfp*$_{11}$]); *oyEx682*[*sra-6*p::*gfp*$_{1-10}$; *unc-122*p::*gfp*]	This work
PY12601	*osm-5*(*oy177*[*osm-5*::*gfp*$_{11}$])	This work
PY12636	*osm-5*(*oy177*[*osm-5*::*gfp*$_{11}$]); *oyEx682*[*sra-6*p::*gfp*$_{1-10}$; *unc-122*p::*gfp*]	This work
PY12637	*osm-3*(*oy156ts*); *osm-5*(*oy177*[*osm-5*::*gfp*$_{11}$]); *oyEx682*[*sra-6*p::*gfp*$_{1-10}$; *unc-122*p::*gfp*]	This work
PY12638	*osm-3*(*p802*); *osm-5*(*oy177*[*osm-5*::*gfp*$_{11}$]); *oyEx682*[*sra-6*p::*gfp*$_{1-10}$; *unc-122*p::*gfp*]	This work
PY12639	*osm-6*(*p811*); *osm-5*(*oy177*[*osm-5*::*gfp*$_{11}$]); *oyEx682*[*sra-6*p::*gfp*$_{1-10}$; *unc-122*p::*gfp*]	This work
PY12640	*daf-19*(*oy204*[*daf-19*::*AID*]); *osm-3*(*oy156ts*); *osm-6*(*oy205*[*osm-6*::*SL2*::*gfp*$_{11}$]); *oyEx682*[*sra-6*p::*gfp*$_{1-10}$; *unc-122*p::*gfp*]; *oyEx830*[*sra-6*p::*TIR1*::*SL2*::*wrmScarlet*]	This work
PY12641	*daf-19*(*oy204*[*daf-19*::*AID*]); *osm-3*(*oy156ts*); *osm-1*(*oy226*[*osm-1*::*SL2*::*gfp*$_{11}$]); *oyEx682*[*sra-6*p::*gfp*$_{1-10}$; *unc-122*p::*gfp*]; *oyEx830*[*sra-6*p::*TIR1*::*SL2*::*wrmScarlet*]	This work
PY12642	*dlk-1*(*km12*); *oyIs14*[*sra-6*p::*gfp*]	This work
PY12643	*dlk-1*(*ju476*); *oyIs14*[*sra-6*p::*gfp*]	This work
PY12644	*oyIs14*[*sra-6*p::*gfp*]; *cebp-1*(*tm2807*)	This work
PY12645	*dlk-1*(*km12*); *kap-1*(*ok676*); *osm-3*(*oy156ts*); *oyIs14*[*sra-6*p::*gfp*]	This work
PY12646	*dlk-1*(*ju476*); *kap-1*(*ok676*); *osm-3*(*oy156ts*); *oyIs14*[*sra-6*p::*gfp*]	This work
PY12647	*dlk-1*(*km12*); *kap-1*(*ok676*); *osm-3*(*oy156ts*); *oyIs14*[*sra-6*p::*gfp*]; *oyEx836*[*sra-6*p::*dlk-1L; unc-122*p::*rfp*]	This work
PY12648	*kap-1*(*ok676*); *osm-3*(*oy156ts*); *oyIs14*[*sra-6*p::*gfp*]; *cebp-1*(*tm2807*)	This work
PY12649	*dlk-1*(*km12*); *kap-1*(*ok676*); *osm-3*(*oy156ts*); *oyIs14*[*sra-6*p::*gfp*]; *cebp-1*(*tm2807*)	This work
PY12650	*kap-1*(*ok676*); *osm-3*(*oy156ts*); *mlk-1*(*km19*); *oyIs14*[*sra-6*p::*gfp*]	This work
PY12651	*kap-1*(*ok676*); *pmk-3*(*ok169*); *osm-3*(*oy156ts*); *oyIs14*[*sra-6*p::*gfp*]	This work
PY12652	*kap-1*(*ok676*); *osm-3*(*oy156ts*); *kgb-1*(*km21*); *oyIs14*[*sra-6*p::*gfp*]	This work

bioRxiv preprint doi: https://doi.org/10.64898/2026.05.15.725580; this version posted July 10, 2026. The copyright holder for this preprint (which was not certified by peer review) is the author/funder, who has granted bioRxiv a license to display the preprint in perpetuity. It is made available under a CC-BY 4.0 International license.

PY12653	*dlk-1*(*km12*); *osm-6*(*oy166*[*osm-6*::*gfp*$_{11}$]); *oyEx682*[*sra-6*p::*gfp*$_{1\text{-}10}$; *unc-122*p::*gfp*]	This work
PHX2595	*dlk-1*(*syb2595*[*dlk-1*::*gfp*$_{11}$])	Yishi Jin
PY12654	*dlk-1*(*syb2595*[*dlk-1*::*gfp*$_{11}$]); *oyEx682*[*sra-6*p::*gfp*$_{1\text{-}10}$; *unc-122*p::*gfp*]; *oyEx832*[*sra-6*p::*eBfp*; *unc-122*p::*rfp*]	This work
PY12655	*dlk-1*(*syb2595*[*dlk-1*::*gfp*$_{11}$]); *kap-1*(*ok676*); *osm-3*(*oy156ts*); *oyEx682*[*sra-6*p::*gfp*$_{1\text{-}10}$; *unc-122*p::*gfp*]; *oyEx832*[*sra-6*p::*eBfp*; *unc-122*p::*rfp*]	This work
PY12667	*cebp-1*(*oy209*[*cebp-1*::*gfp*$_{11}$])	[6]
PY12656	*cebp-1*(*oy209*[*cebp-1*::*gfp*$_{11}$]); *oyEx682*[*sra-6*p::*gfp*$_{1\text{-}10}$; *unc-122*p::*gfp*]	This work
PY12657	*kap-1*(*ok676*); *osm-3*(*oy156ts*); *cebp-1*(*oy209*[*cebp-1*::*gfp*$_{11}$]); *oyEx682*[*sra-6*p::*gfp*$_{1\text{-}10}$; *unc-122*p::*gfp*]	This work
PY12658	*daf-19*(*oy204*[*daf-19::AID*]); *kap-1*(*ok676*); *osm-3*(*oy156ts*); *cebp-1*(*oy209*[*cebp-1*::*gfp*$_{11}$]); *oyEx682*[*sra-6*p::*gfp*$_{1\text{-}10}$; *unc-122*p::*gfp*]; *oyEx830*[*sra-6*p::*TIR1*::*SL2*::*wrmScarlet*]	This work
PY12666	*kap-1*(*ok676*); *osm-3*(*oy156ts*); *oyIs99*[*srh-142*p::*dsRed*; *srh-220*p::*gfp*]	Nikhila Krishnan
PY13100	*oyIs99*[*srh-142*p::*dsRed*; *srh-220*p::*gfp*]	[7]
PY1089	*kyIs104*[*str-1*p::*gfp*]	[3]
PY12659	*kap-1*(*ok676*); *osm-3*(*oy156ts*); *kyIs104*[*str-1*p::*gfp*]	This work
OS2248	*nsIs109*[*F16F9.3*p::*DTA*(*G53E*)	[8]
PY12660	*kap-1*(*ok676*); *osm-3*(*oy156ts*); *kyIs104*[*str-1*p::*gfp*]; *nsIs109*[*F16F9.3*p::*DTA*(*G53E*); *unc-122*p::*gfp*]	This work
PY12661	*kap-1*(*ok676*); *osm-3*(*oy156ts*); *oyIs14*[*sra-6*p::*gfp*]; *nsIs109*[*F16F9.3*p::*DTA*(*G53E*); *unc-122*p::*gfp*]	This work
PY12662	*kap-1*(*ok676*); *osm-3*(*oy156ts*); *oyEx835*[*sra-6*p::*gfp*]	This work
PY12663	*kap-1*(*ok676*); *osm-3*(*oy156ts*); *fig-1*(*tm2079*); *oyEx835*[*sra-6*p::*gfp*]	This work

bioRxiv preprint doi: https://doi.org/10.64898/2026.05.15.725580; this version posted July 10, 2026. The copyright holder for this preprint (which was not certified by peer review) is the author/funder, who has granted bioRxiv a license to display the preprint in perpetuity. It is made available under a CC-BY 4.0 International license.

Table S2, related to all Figures. Gene editing reagents used in this work.

Allele	crRNA Sequence	Donor DNA
daf-19(oy204)	5’ – ACAGAAGACCTGCTTTCTCG – 3’	Plasmid with *AID* sequence and 80 bp homology arms
daf-19(oy210)	5’ – AAGCGGTGGCCCGGAGGCGG – 3’	Plasmid with *gfp* sequence and 400 bp homology arms, inserted into *daf-19(oy204)*
daf-19(oy225)	5’ – CAGTGCAATACCATGTACAG – 3’	Plasmid with *mScarlet-I3* sequence and 80 bp homology arms, inserted into *daf-19(oy210)*
daf-19(oy210 oy242)	5’-CACGACCTTGAATTTTTAAA-3’and 5’-AGTTTCAATCAACCACCTGT-3’	Oligo inserted into *daf-19(oy210)*: 5’-CAGGTCTCGACACGACCTTGAATTTTTAAACATTGCCAGAAATGATGAGGCCAGCTCGCG -3’
osm-6(oy205)	5’ – ATGTGATCACGTTTTTCTAC – 3’	Plasmid with *SL2::gfp$_{11}$* sequence and 60 bp homology arms inserted into *osm-6(oy166)*
osm-1(oy176)	5’ – TGTCATTCAAGTTTTGGTGA – 3’	Plasmid with *gfp$_{11}$* sequence and 60 bp homology arms
osm-1(oy226)	5’ – TCACGCAACGAAATAGCCAA – 3’	Plasmid with *SL2* sequence and 85 bp homology arms, inserted into *osm-1(oy176)*
osm-5(oy177)	5’ – TAAATTATCAATCTGGCAAA – 3’	Plasmid with *gfp$_{11}$* sequence and 75 bp homology arms
osm-5(oy218)	5’ – AGAACCATGTGATCACGATC – 3’	Plasmid with *SL2* sequence and 60 bp homology arms, inserted into *osm-5(oy177)*

bioRxiv preprint doi: https://doi.org/10.64898/2026.05.15.725580; this version posted July 10, 2026. The copyright holder for this preprint (which was not certified by peer review) is the author/funder, who has granted bioRxiv a license to display the preprint in perpetuity. It is made available under a CC-BY 4.0 International license.

REFERENCES

1. Jin, Y., and Zheng, B. (2019). Multitasking: dual leucine zipper-bearing kinases in neuronal development and stress management. Annu Rev Cell Dev Biol *35*, 501-521.
2. Byrne, A.B., and Hammarlund, M. (2017). Axon regeneration in *C. elegans*: Worming our way to mechanisms of axon regeneration. Exp Neurol *287*, 300-309.
3. Troemel, E.R., Chou, J.H., Dwyer, N.D., Colbert, H.A., and Bargmann, C.I. (1995). Divergent seven transmembrane receptors are candidate chemosensory receptors in *C. elegans*. Cell *83*, 207-218.
4. Philbrook, A., O'Donnell, M.P., Grunenkovaite, L., and Sengupta, P. (2024). Cilia structure and intraflagellar transport differentially regulate sensory response dynamics within and between *C. elegans* chemosensory neurons. PLoS Biol *22*, e3002892.
5. Khan, M., Hartmann, A.H., O'Donnell, M.P., Piccione, M., Pandey, A., Chao, P.-H., Dwyer, N.D., Bargmann, C.I., and Sengupta, P. (2022). Context-dependent reversal of odorant preference is driven by inversion of the response in a single sensory neuron type. PLoS Biol *20*, e3001677.
6. Bates, S.G., Harris, N., and Sengupta, P. (2026). A repressive regulatory cascade shapes temporal patterning of activity-regulated gene expression in a defined sensory neuron type. bioRxiv, https://doi.org/10.64898/62026.64805.64814.725236.
7. Krishnan, N., Lawson, H., Leslie, S., Lu, Y.-M., Wexler, L., Heiman, M.G., and Sengupta, P. (2026). Stereotypical interciliary contacts in a *C. elegans* sense organ. bioRxiv, https://doi.org/10.64898/62026.64804.64810.717756.
8. Bacaj, T., Tevlin, M., Lu, Y., and Shaham, S. (2008). Glia are essential for sensory organ function in *C. elegans*. Science *322*, 744-747.
