# Supplementary material for: Neuron-intrinsic and glial pathways regulate sensory cilia regeneration in adult C. elegans
Source: bioRxiv. 2026 May 17:2026.05.15.725580. Preprint. [Version 1] doi: 10.64898/2026.05.15.725580 (PMC13192683; doi:10.64898/2026.05.15.725580)
Supplement: Supplementary file 1 [file media-1.pdf]

SUPPLEMENTAL FIGURE LEGENDS

Figure S1. ASH cilia truncate and regrow rapidly in the adult.

Quantification of ASH cilia length in wildtype (black) and *kap-1(ok676); osm-3(oy156ts)* (blue) mutants at the indicated temperature shift conditions. Each circle is the length of a single ASH cilium. ***: different at $P<0.001$ from unshifted within each genotype; ns: not significant (one-way ANOVA with Tukey's multiple comparison test). Errors are SEM. n≥44 each; 3 independent experiments.

Figure S2. DAF-19 is required for cilia regeneration but is dispensable for maintenance.

A) Percentage of animals of the indicated genotypes in which a subset of ciliated neurons including ASH/ASI are filled with dye. n>300 animals each; 3 independent experiments.

B) Representative images of endogenously tagged DAF-19::AID::GFP expression in ASH/ASI neurons in the absence or presence of auxin as indicated. These neurons also express *sra-6*p*::TIR1::SL2::mScarlet* from an extrachromosomal array. ASH/ASI soma are indicated with dashed circles. Scale bar: 5 µm.

C) (Top) Schematic of experimental conditions for ASH cilia length quantifications (bottom). Each circle is the length of a single ASH cilium. ***: different at $P<0.001$ from unshifted or between indicated; ns: not significant (one-way ANOVA with Tukey's multiple comparison test). Errors are SEM. n≥36 each; 3 independent experiments.

Figure S3. IFT protein levels in ASH soma are altered upon cilia truncation and regeneration.

A) Schematic of endogenously tagged translational reporter.

B-D) Quantification of fluorescence intensities in ASH/ASI cilia (left) and soma (right) of wildtype (black) or *osm-3(oy156ts)* (blue) animals expressing the indicated IFT translational reporters in the shown temperature shift conditions. Each circle is the combined value from ASH/ASI cilia (left) or soma (right) in an amphid organ. * and **: different at $P<0.05$ and 0.01, respectively, from unshifted within each genotype or between indicated; ns: not significant (one-way ANOVA with Tukey's multiple comparison test). Errors are SEM. n≥39 each; >3 independent experiments.

E) Quantification of fluorescence intensities of the *osm-6::SL2::split-gfp* transcriptional reporter in ASH/ASI soma in wildtype and the indicated mutants Each circle is the combined value from ASH/ASI soma in an amphid organ. ***: different at $P<0.01$ from unshifted; ns: not significant (one-way ANOVA with Tukey's multiple comparison test). Errors are SEM. n≥84 each; >3 independent experiments.

Figure S4. DLK-1 and CEBP-1 are necessary for efficient cilia regeneration.

A) Quantification of ASH cilia length in the shown genetic backgrounds Each circle is the length of a single ASH cilium. ** and ***: different at $P<0.01$ and 0.001 from wildtype; ns: not significant (one-way ANOVA with Tukey's multiple comparison test). Errors are SEM. n≥35 each; 2 independent experiments.

B,E) Quantification of ASH cilia length in the shown genetic backgrounds at the indicated temperature shift conditions. Each circle is the length of a single ASH cilium. ***: different at $P<0.001$ from unshifted within each genotype; ns: not significant (B: t-test, E: one-way ANOVA with Tukey's multiple comparison test). Errors are SEM. n≥44 each; 3 independent experiments.

C) Histogram (left) and quantification (right) of velocities of reconstituted endogenously tagged OSM-6::split-GFP in ASH cilia in wildtype (top) and *dlk-1(km12)* animals. n≥24 animals per genotype; 3 independent experiments. ns: not significant (t-test).

D) Known DLK-1-regulated signaling pathways. Adapted from [80,125].

Figure S5. Cilia morphology is altered in the absence of glia.

Representative images of ASH (A) and AWB (B) cilia in the indicated genetic backgrounds and temperature shift conditions. Yellow/white arrowheads: cilia base/cilia tip. Scale bars: 5 µm.

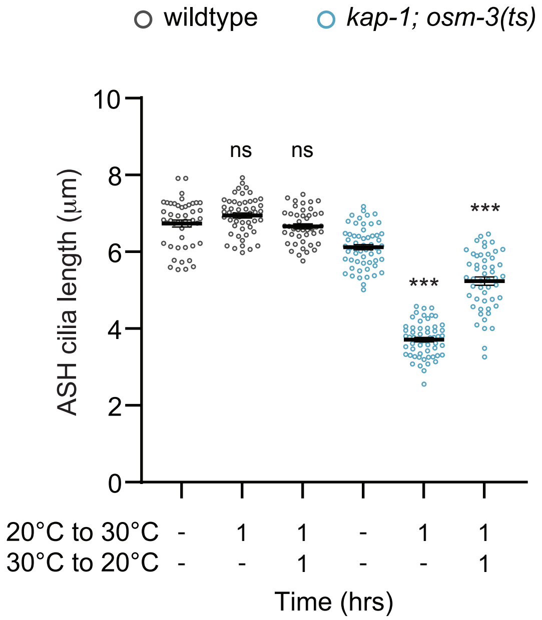

Judge et al., Figure S1

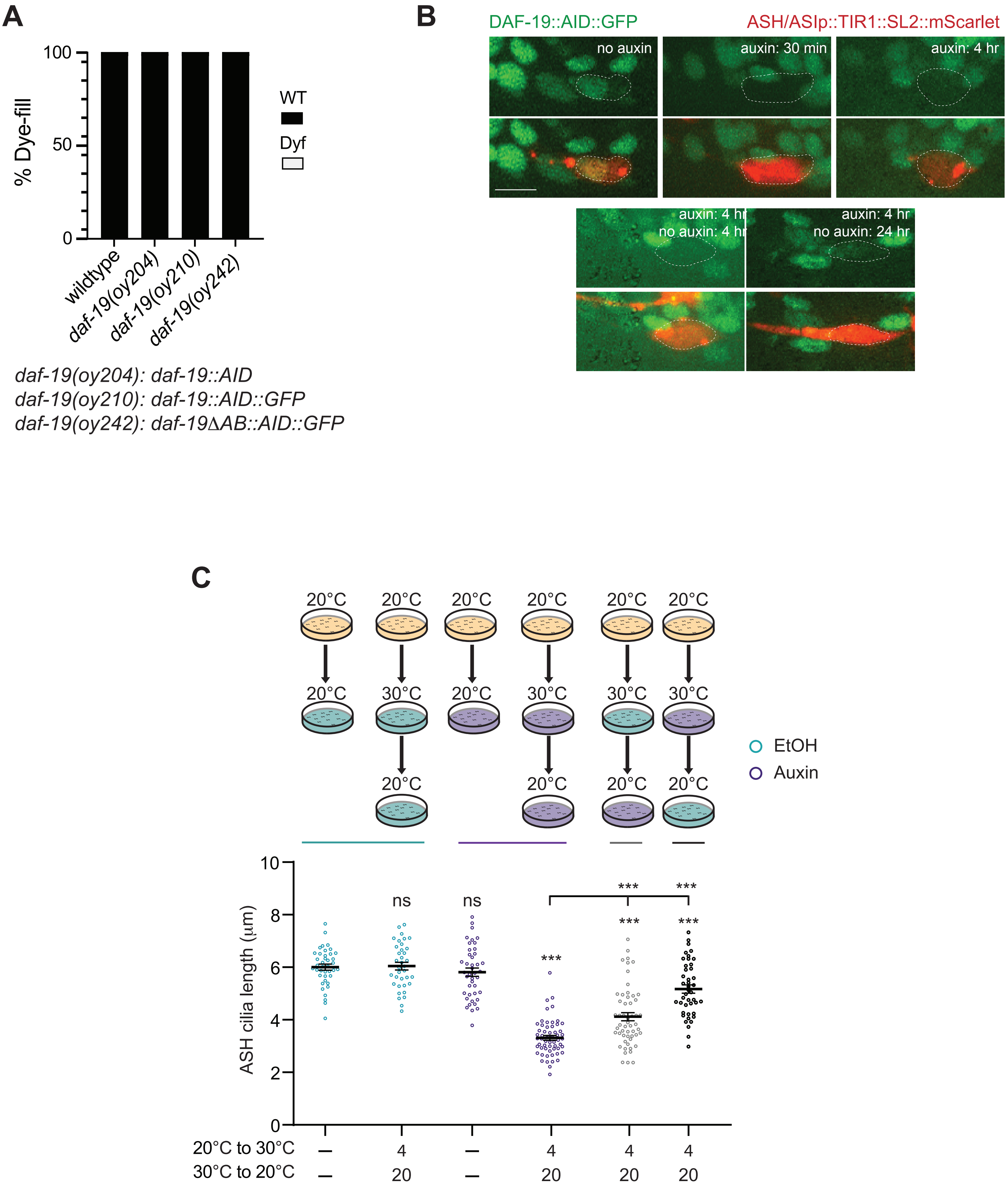

Judge et al., Figure S2

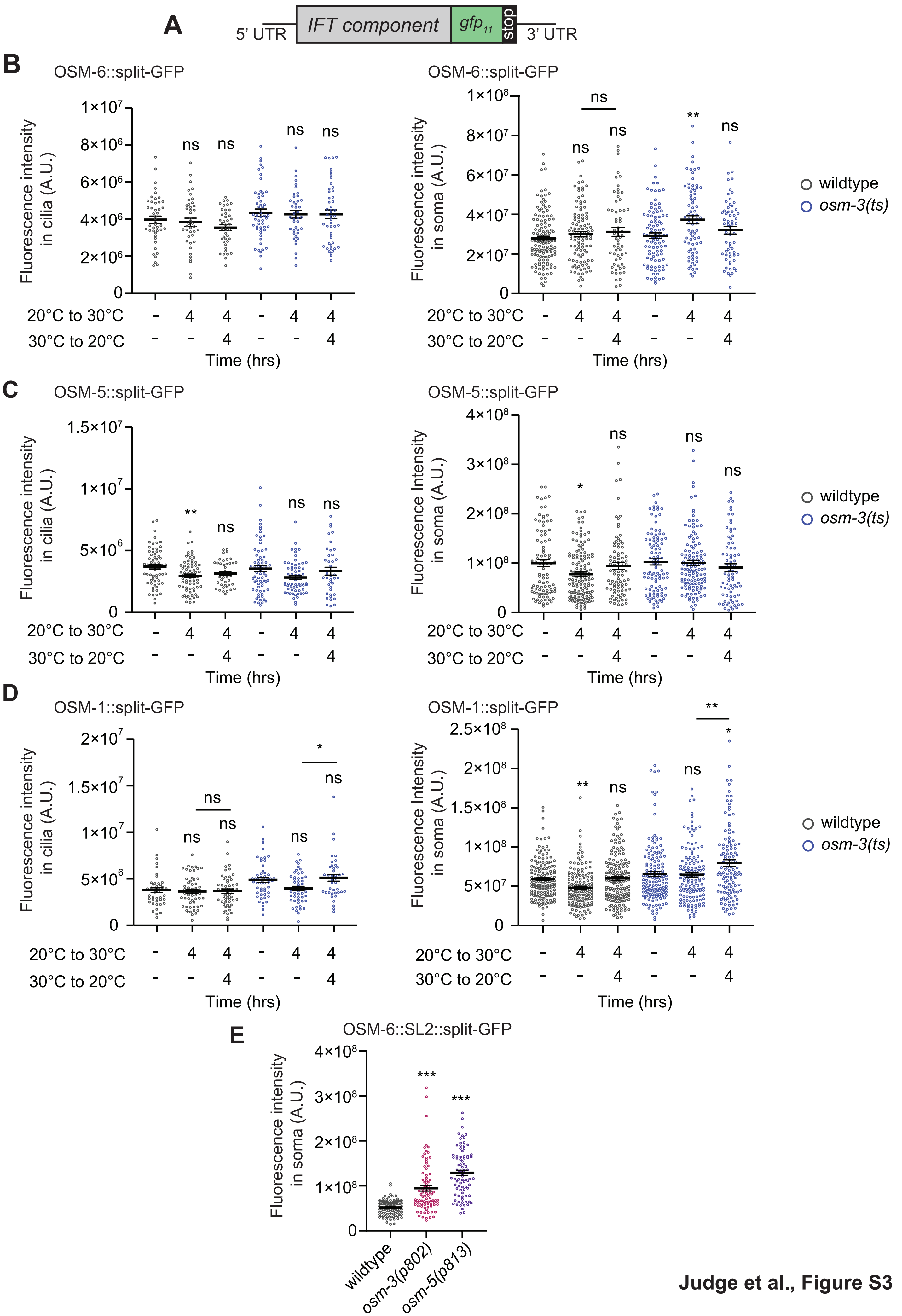

Judge et al., Figure S3

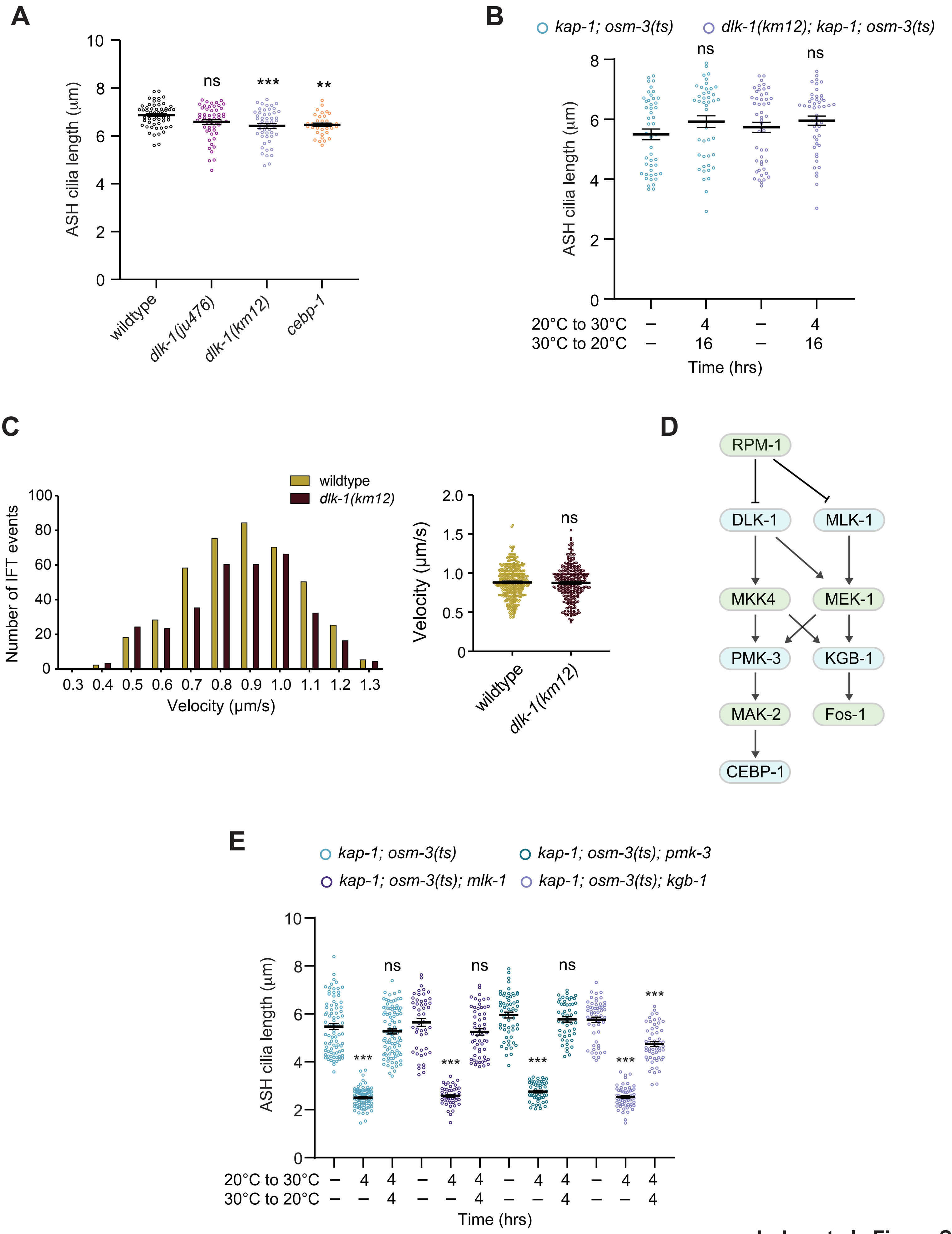

Judge et al., Figure S4

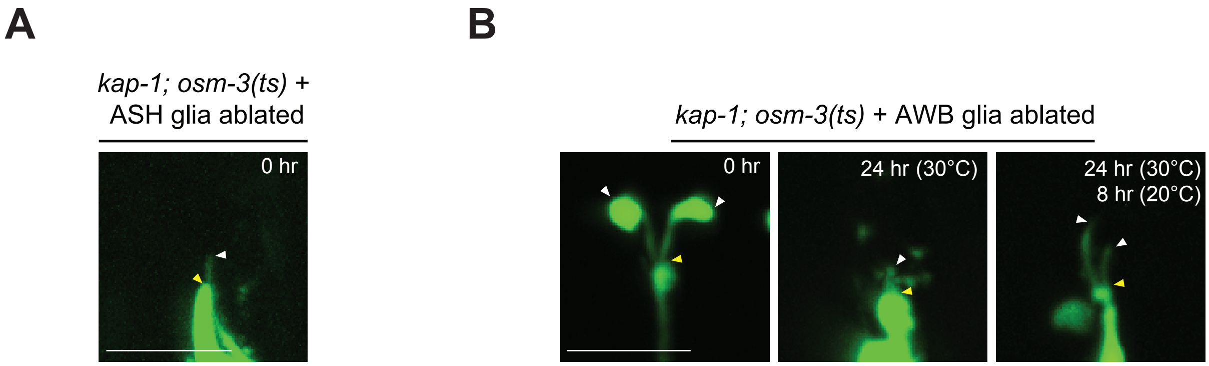

Judge et al., Figure S5
